# Triplet Acceptors with a D‐A Structure and Twisted Conformation for Efficient Organic Solar Cells

**DOI:** 10.1002/anie.202006081

**Published:** 2020-06-09

**Authors:** Linqing Qin, Xingzheng Liu, Xin Zhang, Jianwei Yu, Lei Yang, Fenggui Zhao, Miaofei Huang, Kangwei Wang, Xiaoxi Wu, Yuhao Li, Hao Chen, Kai Wang, Jianlong Xia, Xinhui Lu, Feng Gao, Yuanping Yi, Hui Huang

**Affiliations:** ^1^ Center of Materials Science and Optoelectronics Engineering College of Materials Science and Opto-Electronic Technology &, CAS Center for Excellence in Topological Quantum Computation &, CAS Key Laboratory of Vacuum Physics University of Chinese Academy of Sciences Beijing 100049 P. R. China; ^2^ Department of Physics, Chemistry and Biology (IFM) Linköping University 58183 Linköping Sweden; ^3^ Key Laboratory of Luminescence and Optical Information Ministry of Education School of Science Beijing Jiaotong University Beijing 100044 P. R. China; ^4^ Beijing National Laboratory for Molecular Sciences CAS Key Laboratory of Organic Solids, CAS Research/Education Center for Excellence in Molecular Sciences Institute of Chemistry Chinese Academy of Sciences Beijing 100190 P. R. China; ^5^ State Key Laboratory of Advanced Technology for Materials Synthesis and Processing Center of Smart Materials and Devices School of Chemistry, Chemical Engineering and Life Science Wuhan University of Technology Wuhan 430070 P. R. China; ^6^ Department of Physics The Chinese University of Hong Kong New Territories Hong Kong P. R. China

**Keywords:** D-A structures, long lifetime excitons, organic solar cells, triplet acceptors, twisted conformation

## Abstract

Triplet acceptors have been developed to construct high‐performance organic solar cells (OSCs) as the long lifetime and diffusion range of triplet excitons may dissociate into free charges instead of net recombination when the energy levels of the lowest triplet state (T_1_) are close to those of charge‐transfer states (^3^CT). The current triplet acceptors were designed by introducing heavy atoms to enhance the intersystem crossing, limiting their applications. Herein, two twisted acceptors without heavy atoms, analogues of Y6, constructed with large π‐conjugated core and D‐A structure, were confirmed to be triplet materials, leading to high‐performance OSCs. The mechanism of triplet excitons were investigated to show that the twisted and D‐A structures result in large spin–orbit coupling (SOC) and small energy gap between the singlet and triplet states, and thus efficient intersystem crossing. Moreover, the energy level of T_1_ is close to ^3^CT, facilitating the split of triplet exciton to free charges.

## Graphical abstract

**Triplet materials** are designed by introducing heavy atoms to enhance spin–orbit coupling or constructing donor and acceptor units with a twisted conformation to reduce Δ*E*_ST_. However, the twisted materials have not been applied in solar cells due to weak absorption and low charge‐transport mobilities. Now two nonplanar acceptors with large π‐conjugated core were constructed that achieved over 15 % efficiency.

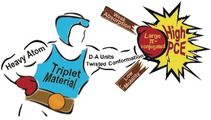

## Introduction

OSCs have rapidly developed over the past decades owing to their advantages of low cost, flexibility, and light weight.^1^ With the efforts on the materials development and device engineering, the power conversion efficiency (PCE) of OSCs have now reached over 17 %.^2^ The working mechanism of OSCs includes photon absorption and exciton generation, exciton diffusion, exciton split and charge generation, charge transport, and charge collections. Thus, the PCE of OSCs equals to the time of the efficiency of each step. Obviously, one of the important strategies of improving the efficiency of OSCs is to increase the exciton diffusion distance. Although triplet excitons can travel longer than singlet excitons, the role and mechanisms of triplet excitons in OSCs are still elusive.^3^ Thus, it is of importance to develop triplet materials to investigate the mechanisms.

The generation of T_1_ state depends on the enhancement of the intersystem crossing (ISC) from the lowest singlet state (S_1_) to T_1_.^4^ According to the perturbation theory, the rate constant (*k*_ISC_) of ISC is given by Equation (1):

(1) kISC∝⟨1Ψ|H^SO|3Ψ⟩/exp(ΔE2ST)

where ⟨^1^ *Ψ*|*Ĥ*_SO_|^3^ *Ψ*⟩ is the spin–orbit coupling matrix element, *Ĥ*_SO_ is the spin–orbit coupling Hamiltonian, and Δ*E*_ST_ is the energy gap between the singlet and triplet states. This equation suggests that large spin–orbit coupling value and small Δ*E*_ST_ can afford high *k*_ISC_. Incorporation of heavy atoms into the π‐conjugated systems can enhance the spin–orbit coupling, facilitating the ISC to generate triplet excitons. Various triplet materials containing heavy atoms have been developed for high‐performance OSCs. For example, Yang et al. reported a triplet platinum porphyrine‐based donor materials for OSCs, affording an efficiency of 2.1 %,^5^ while Huang and co‐workers introduced iridium into the backbone of PTB7 to significantly improve the efficiency of OSCs with over 40 %, affording an efficiency of 8.71 %.^6^ In 2017, Huang et al. reported the first tellurophene‐based triplet acceptors for OSCs.^7^ The diffusion distances of triplet excitons were estimated to be 30 nm, which is comparable to other triplet fullerene derivatives. As a result, an efficiency of 7.52 % was achieved, which is much higher than that of the thiophene analogue based OSCs. According to Equation (1), reducing Δ*E*_ST_ is another important method to increase *k*_ISC_ to achieve triplet materials. One of important strategies to minimize Δ*E*_ST_ is combining nonplanar donor (D) and acceptor (A) units in conjugated systems, which has been employed to construct various organic room‐temperature phosphorescence^8^ and thermally activated delayed fluorescence materials.^9^ However, these type of triplet materials have never been used for OSCs since their twist structures usually led to weak light absorption intensities and low charge transport mobilities, which is detrimental for efficiency of OSCs.

**Figure 1:**
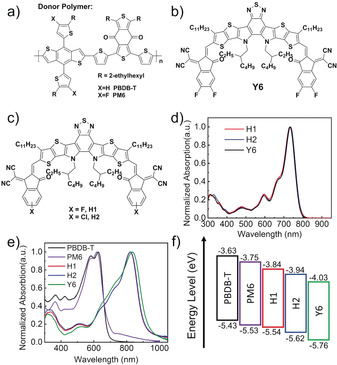
The molecular structures of a) donor polymer PBDB‐T and PM6, b) Y6, and c) H1, H2. d) Normalized UV‐vis absorption spectra of acceptors as solutions. e) Normalized UV‐vis absorption spectra of donors and acceptors as thin films. f) Energy levels of PBDB‐T, PM6, H1, H2 and Y6 obtained from CV.

Herein, two twisted‐conformation molecular semiconductors with A‐D‐A′‐D‐A structure (H1 and H2), analogues of Y6,2c, 10 were constructed based on a large π‐conjugated fused core (Figure 1 b,c), which were shown to be triplet acceptors with strong light absorption, supported by steady and transient photoluminescence, and absorption spectroscopy, electron paramagnetic resonance (EPR), magneto‐photocurrent (MPC), and time‐dependent density function theory (TD‐DFT). The results revealed that the D‐A structure with nonplanar conformation reduced the Δ*E*_ST_ and facilitated the ISC, yielding triplet excitons efficiently. Moreover, the energy level of the T_1_ state is rather close to the ^3^CT state, which is beneficial for the split of triplet excitons to free charges. Finally, the large π‐conjugated fused core of the acceptors afforded strong light absorption, which is beneficial for the photocurrents. As a result, high‐performance OSCs based on these acceptors were fabricated to afford efficiencies of over 15 %, demonstrating that the triplet excitons were generated and split in the blend films to contribute to the PCE, supported by magneto‐photocurrent and transient spectroscopy.

## Results and Discussion

The compounds H1 and H2, synthesized through Knoevenagel condensation (Supporting Information, Scheme S1), were fully characterized with ^1^H and ^13^C NMR spectroscopy and elementary analysis. Figure 1 d shows that the absorption spectra of Y6, H1, and H2 in solution were rather close to each other, in the range of 400–800 nm with an absorption peak at 735 nm, while the absorption peaks of the thin films demonstrated around 90 nm red‐shift (Figure 1 e), indicating that Y6, H1, and H2 possess strong intermolecular interactions and electronic coupling in the solid state.10a The absorption coefficient were estimated to be 9.95×10^4^ cm^−1^, 1.01×10^5^ cm^−1^, and 1.03×10^5^ cm^−1^ for H1, H2, and Y6 films, respectively (Supporting Information, Figure S6), which can be attributed to the large π‐conjugation.

The electrochemical properties of these acceptors were investigated by cyclic voltammetry (CV; Supporting Information, Figure S8). According to the equation *E*_HOMO/LUMO_=−*e*(*E*_onset,ox/red_+4.71 eV), the energy levels of HOMO/LUMO for H1, H2, and Y6 are calculated to be −5.54/−3.84 eV, −5.62/−3.94 eV, and −5.76/−4.03 eV, respectively (Figure 1 f). Obviously, Y6 possesses the lowest energy levels owing to the strong electron‐withdrawing properties of four fluorine atoms in the end groups.

**Figure 2:**
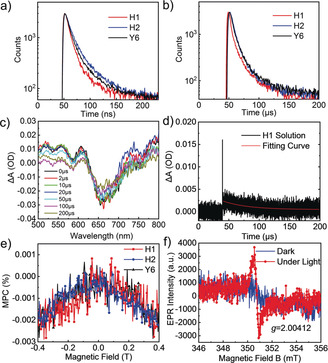
a) Time‐resolved transient photoluminescence decay traces of H1, H2, and Y6 at 298 K. b) Time‐resolved transient photoluminescence decay traces of H1, H2, Y6 at 77 K. c) Transient absorption spectrum of H1 in degassed chloroform. d) Decay traces of H1 probed at 550 nm. e) Magneto‐photocurrent of H1, H2, and Y6 pristine films; the device structure is ITO/ZnO/prinstine film/MoO_3_/Ag. f) Electron paramagnetic resonance spectra of H1 in dark and under light conditions.

Time‐resolved transient photoluminescence was employed to estimate the excited state lifetime of these acceptors. Figure 2 a showed that Y6, H1, and H2 in 2‐methylfuran solution possess a short excited‐state lifetime of 11.67 ns, 13.36 ns, and 10.41 ns at 298 K, respectively. However, the lifetime increased sharply to 6.07 μs, 8.15 μs, and 7.66 μs, respectively, when the solution samples were cooled down to 77 K (Figure 2 b). This observation suggested that ISC is efficient in these acceptors, generating a large amount of triplet excitons.^11^

Transient absorption spectroscopy is another effective method to investigate the dynamics of triplet excitons. As shown in Figure 2 c, transient spectra in degassed chloroform solution share two photoinduced absorption (PIA) bands at around 530 nm and 850 nm, standing for the kinetic process of excited state absorption (ESA),^12^ and a strong ground state bleaching (GSB) peak at around 660 nm, consistent with the solution absorption spectra. The decay traces of H1, H2, and Y6 are shown in Figure 2 d and the Supporting Information, Figure S9, and their lifetimes were evaluated to be 42 μs, 55 μs, and 41 μs, respectively, which is consistent with the lifetime measured by time‐resolved transient photoluminescence. Thus, the ESA peaks were reasonably assigned to the upper transitions from T_1_ to T_*n*_ of these acceptors.^13^

TD‐DFT calculation was applied to probe the generation of the triplet excitons. Two halves of the molecules share a dihedral angel of 16.87°, 16.90°, and 16.88° for H1, H2, and Y6, respectively (Supporting Information, Figures S10–S12), suggesting their twisted structures, which may be beneficial for reducing the Δ*E*_ST_.^14^ As discussed above, the ISC process is decided by Δ*E*_ST_ and spin–orbit coupling constants, where a small Δ*E*_ST_ and a large spin–orbit coupling constant may lead to an efficient ISC. Detailed calculated data of excited states energy levels and spin–orbit coupling constants are summarized in the Supporting Information, Tables S1–S6. The Δ*E*_ST_ of H1 between S_2_ and T_3_ is only 0.0672 eV, and the spin–orbit coupling constant of around 0.1 between S_2_ and T_3_ is exhibited. These data combined are comparable to afford a high *k*_ISC_,^15^ thus providing an efficient ISC channel.

Magneto‐photocurrent experiments were performed to investigate the triplet properties of these acceptors.^16^ Magneto‐photocurrent can be defined as MPC=(*I*(B)−*I*(0))/*I*(0),^17^ in which *I*(B) and *I*(0) are the photocurrent in the presence and absence of magnetic field, respectively. The results of the pristine acceptor films are presented in Figure 2 e. The magnetic field can manipulate singlet‐to‐triplet ratio through Larmor precession, which will influence photocurrent effectively.^18^ All three acceptor films exhibited a negative signal as the magnetic field strength increases, which indicate triplet excitons are more likely to be produced at excited states, and the effects originate from the triplet‐charge reaction,^19^ which decreased photocurrent.

Electron paramagnetic resonance measurements can be applied to detect signals and analyze information of the states and excitons because it is a spin‐sensitive technique.^20^ The dark and under light electron paramagnetic resonance spectra were shown in Figure 2 f and the Supporting Information, Figure S13. An electron paramagnetic resonance signal was observed under light around 351 mT for these acceptors, suggesting their paramagnetic properties,^21^ and the corresponding *g*‐factors for H1, H2, and Y6 are 2.00412, 2.00499, and 2.00462 respectively. Considering the fingerprints and the magnetic field width of approximately 1.5 mT, the electron paramagnetic resonance signal can be attributed to triplet CT state (^3^CT) polaron pairs,^22^ which shall be transformed from ^1^CT polaron pairs through ISC. These results further illustrated the triplet nature of these acceptors.

**Figure 3:**
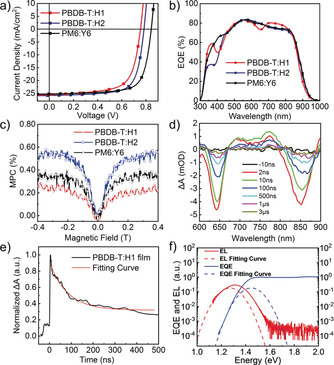
a) *J*–*V* curves and b) EQE curves of the H1, H2 and Y6 based OSCs. c) Magneto‐photocurrent of H1, H2 and Y6 based OSCs. d) Transient absorption spectrum of PBDB‐T:H1 blend film. e) Decay traces of PBDB‐T:H1 blend film probed at 770 nm. f) EL and EQE spectra of PBDB‐T:H1 based devices.

PBDB‐T and PM6 were chosen as the donors to couple with the three acceptors since they have complimentary light absorption and matched energy levels. OSCs based on H1, H2, and Y6 were fabricated with a conventional structure of ITO/PEDOT:PSS/active layer/PDINO/Al to study their performance on OSCs. The typical current density‐voltage (*J*–*V*) curves and the external quantum efficiency (EQE) curves are shown in Figure 3 a,b, and the device performance parameters are summarized in Table 1. After preliminary optimization, a best PCE of 14.16 % for PBDB‐T:H1 devices was obtained with a *V*_OC_ of 0.76 V, a *J*_SC_ of 25.74 mA cm^−2^ and a FF of 71.40 %, which are close to reported results of Y14.^23^ When H1 is replaced by H2, the PCE climbed over 15 % with a *V*_OC_ of 0.79 V, a *J*_SC_ of 25.82 mA cm^−2^, and a FF of 73.86 %. The enhancement of *J*_SC_ can be ascribed to the stronger absorptivity, while the enhancement of FF may be attributed by the better crystallinity of the chlorine atom,^24^ which lead to a higher electron mobility. The improvement of *V*_OC_ is however abnormal, since H2 have a lower LUMO energy level compared to H1, which will be discussed later in the following parts. These results suggested that both H1 and H2 can be applied as high‐performance acceptors for efficient OSCs. However, the PBDB‐T:Y6 based solar cells only afforded a relatively low efficiency of 9.88 % (Supporting Information, Table S7). Thus, PM6 was used as the donor to couple with Y6, affording a high performance OSCs with a best PCE of 15.35 % (a *V*_OC_ of 0.83 V, a *J*_SC_ of 25.24 mA cm^−2^ and a *FF* of 74.07 %), which is comparable to the reported results.10a

**Table 1:** Detailed photovoltaic parameters of the OPV cells based on ten devices.

Devices	*V*_OC_ [V]	*J*_SC_ [mA cm^−2^]	FF	PCE [%]
PBDB‐T:H1	0.76±0.01	25.74±0.21	0.71±0.02	14.06(13.70±0.13)
PBDB‐T:H2	0.79±0.01	25.82±0.19	0.73±0.01	15.12(14.89±0.19)
PM6:Y6	0.83±0.01	25.24±0.25	0.74±0.02	15.35(15.10±0.21)

The EQE curves (Figure 3 b) illustrated that the devices have a broad photoresponse range from 300 nm to 950 nm, which is consistent with the UV/Vis absorption of the blend films. All devices exhibited a high EQE of over 70 % from 450 nm to 850 nm, and the maximum EQE value are close to 85 %, suggesting an efficient process of photoelectron conversion for all devices. The integrated *J*_SC_ results calculated for these devices are 24.92, 25.13, and 24.56 mA cm^−2^ for H1, H2, and Y6 based devices, respectively, which are close to the *J*_SC_ from *J*–*V* measurements.

The magneto‐photocurrent on PBDB‐T:H1, PBDB‐T:H2, and PM6:Y6 OSC devices were then measured (Figure 3 c). All of the measurements exhibit a positive signal, and the magneto‐photocurrent are gradually increased with the rising field strength. In fact, since the electron and hole dissociation and recombination are spin‐dependent in the photovoltaic process for OSCs,^25^ such a line‐shape denotes that the dominant mechanism behind the increase of photocurrent is the exciton dissociation at charge transfer states owing to the increase of the magnetic field strength.^26^ Judging from Figure 3 c, the full width at half maximum (FWHM) of magneto‐photocurrent for PBDB‐T:H2 based devices seems to be the narrowest in comparison to the rest. It also exhibits the largest magneto‐photocurrent effect among these three, suggesting that the exciton dissociation at charge transfer states is more efficient in PBDB‐T:H2 based solar cells. The results are consistent with the device performance parameters given in Table 1, where the PBDB‐T:H2 based solar cells produce the highest *J*_SC_.

Transient absorption spectra experiments were employed to further analyze the dynamics of excitons in the blend films. All blend films were sealed with PMMA film to avoid oxygen quenching in air. The films were excited by a 600 nm laser beam (45 μW). Figure 3 d showed two strong GSB peaks at around 640 nm and 850 nm, which are consistent with absorption peaks of the pristine films of donors and acceptors, respectively. Moreover, a wide range of PIA band is observed between these two peaks, which stands for the charge‐transfer process.^12^ The decay lifetime was investigated for the strongest signal at 770 nm, and the decay traces (Figure 3 e; Supporting Information, Figure S15) demonstrate that the lifetime has dropped to 65 ns, 30 ns, and 45 ns for H1, H2, and Y6 in blend films, respectively. These excitons can usually be classified as triplet excitons, since singlet excitons will not be able to possess such a long lifetime.^27^ Considering the long lifetime of the excitons, geminate recombination were suppressed in the blend film,^28^ while non‐geminate recombination was observed to be rather weak, based on the light intensity dependence measurements (Supporting Information, Figure S18). Since geminate and non‐geminate recombination is both negligible in the blend film, these combined observations indicate that triplet excitons can be generated and split into free charges at the D/A interfaces.^7^

EQE and electroluminescence (EL) quantum efficiency were used to measure the energy losses and state energy levels to further understand the mechanism of the photovoltaic devices. The energy loss (Δ*E*) was divided into three different parts according to Equation (2):^28^

(2) ΔE=qΔV=Eg-qVOC=(Eg-qVSQOC)+(qVSQOC-qVradOC)+(qVradOC-qVOC)=(Eg-qVSQOC)+qΔVrad,belowgapOC+qΔVnon-radOC=ΔE1+ΔE2+ΔE3

in which VSQOC stands for the maximum voltage under the SQ limit, VradOC stands for the open‐circuit voltage when there is only radiative recombination existing, ΔVrad,belowgapOC stands for the energy loss for radiative recombination due to below gap absorption, and ΔVnon-radOC stands for the voltage loss of non‐radiative recombination.^29^ Δ*E*_1_ is the radiative recombination energy loss above the band gap, which is unavoidable in all kinds of solar cells. Δ*E*_2_ is the radiative energy loss below the band gap, owing to the nonstop function absorption. Δ*E*_3_ is the non‐radiative energy loss, which comes from the non‐radiative recombination.^30^ It could be calculated by Equation (3):

(3) ΔE3=qΔVrad,belowgapOC=-kTln(EQEEL)

The detailed data of the energy loss in devices of these two acceptors are enlisted in Table 2. The optical band gap (*E*_g_) can be extracted from EQE spectra.^31^ Both devices exhibited very close Δ*E*_1_ value of 0.26 eV according to Shockley–Queisser (SQ) theory.^32^ As a result, VSQOC of H1 and H2 devices can be evaluated to be 1.15 V and 1.16 V, respectively. Δ*E*_2_ can be derived from EQE spectra, and both devices have a steep curve of high sensitive EQE (Figure 3 f; Supporting Information, Figure S20), which shows that H1 and H2 possess a similar low Δ*E*_2_ value of 0.07 eV and 0.06 eV, respectively.^33^ According to Equation (2), Δ*E*_3_ was estimated based on EQE_EL_. H1 and H2 devices process EQE_EL_ of 2.8×10^−6^ and 7.4×10^−6^, respectively (Supporting Information, Figure S21), which are relatively high in OSCs.^29^ Thus, the corresponding Δ*E*_3_ are 0.33 eV and 0.31 eV for H1 and H2, respectively. This observation supported that the H1 based solar cells unusually possess a smaller *V*_OC_ than H2 based ones, although H1 has a higher LUMO energy level than H2.10c

**Table 2:** Detailed data of *V*_OC_ loss and excited states energy levels of the PBDB‐T:H1 and PBDB‐T:H2 based devices.

Material	*E*_g_ [eV]	Δ*E* [eV]	qVSQOC [eV]	Δ*E*_1_ [eV]	Δ*E*_2_ [eV]	Δ*E*_3_ [eV]	S_1_ [eV]	T_1_ [eV]	CT [eV]
H1	1.41	0.66	1.15	0.26	0.07	0.33	1.41	1.06	1.35
H2	1.42	0.63	1.16	0.26	0.06	0.31	1.42	1.08	1.38

The energy levels of the CT state and excited state were evaluated to understand the roles of these states in the photovoltaic performances. We take S_1_ to be equivalent to *E*_g_ here, and the energy levels of S_1_ of H1 and H2 are thus determined to be 1.41 eV and 1.42 eV, respectively. The T_1_ energies of H1 and H2 are 1.06 eV and 1.08 eV, respectively, based on the emission band of films occurred at low temperature (Supporting Information, Figure S22).^34^ The small Δ*E*_ST_ between S_1_ and T_1_ may strongly promote ISC process,^35^ resulting in large amounts of triplet excitons in the system. Moreover, through fitting the low‐energy shoulder of the EL and EQE spectra,^36^ CT state energy levels of H1 and H2 are determined to be 1.35 eV and 1.38 eV,^31^ which is close to the T_1_ state. Consequently, triplet excitons may be allowed to form ^3^CT, which provides sufficient time to subsequently dissociate excitons into free charges and thus contributes to the photovoltaic performance.

To further understand the effect of the end‐groups on the fill factors, charge transport mobilities of blend films were investigated by space charge limit current (SCLC) method (Supporting Information, Figure S23). The hole mobilities for H1, H2, and Y6 based blend films are 5.17×10^−4^, 5.24×10^−4^, and 5.76×10^−4^ cm^2^ V^−1^ s, respectively, while the electron mobilities are 3.47×10^−4^, 4.21×10^−4^, and 3.89×10^−4^ cm^2^ V^−1^ s, respectively. The electron mobility of H2 based blend films is higher than that of H1 based ones, which may be because the H2 possesses stronger accumulation with its chlorine end‐groups, resulting more balanced hole/electron mobility and a higher FF.

**Figure 4:**
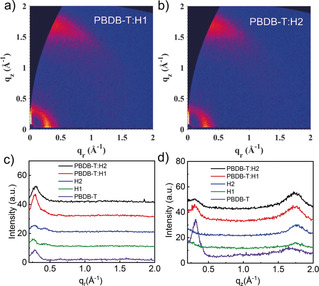
a),b) 2D GIWAXS patterns of a) PBDB‐T:H1 and b) PBDB‐T:H2 blend films. c),d) Intensity profiles along the c) in‐plane and d) out‐of‐plane directions.

Grazing‐incidence wide‐angle X‐ray scattering (GIWAXS) was used to probe the molecular packing of neat PBDB‐T, H1, and H2 pristine films and PBDB‐T:H1 and PBDB‐T:H2 blend film, and the results of the 2D GIWAXS patterns are shown in Figure 4 and the Supporting Information, Figure S24. Neat PBDB‐T film exhibited strong crystallinity with a (100) lamellar peak both in the out‐of‐plane (OOP) direction at *q*=0.28 Å^−1^ (*d*≈22.5 Å) and in the in‐plane (IP) direction at *q*=0.40 Å^−1^ (*d*≈15.8 Å). The polymer donor film also presents a π–π peak in the OOP direction at *q*=1.65 Å^−1^ (*d*≈3.8 Å). Neat H1 and H2 film both have a π–π stacking peak in the OOP direction at around *q*=1.75 Å^−1^ (*d*≈3.60 Å), and signal of H2 is even much stronger, which could be because the chlorine end‐group processes better accumulation than fluorine end‐group.10c Also, two peaks in the IP direction were observed in these two neat films at around *q*=0.25 Å^−1^ (*d*≈25.2 Å) and *q*=0.42 Å^−1^ (*d*≈15.0 Å). The peak at *q*=0.25 Å^−1^ (*d*≈25.2 Å) can be identified as lamellar peak, while the peak at *q*=0.42 Å^−1^ (*d*≈15.0 Å) may be ascribed to the backbone ordering owing to π–π stacking of the end‐group. The annealed blend film of PBDB‐T:H1 and PBDB‐T:H2 showed a strong peak at *q*=1.75 Å^−1^ (*d*≈3.60 Å) in the OOP direction, which is consistent with the π–π peak of neat acceptor films, and the π–π peak of neat donor film disappeared in the blend films, suggesting crystallinity of PBDB‐T has been weakened. Meanwhile, in these blend films, we can observe an enhancement of the lamellar peak of the polymer in the IP direction at *q*=0.28 Å^−1^ (*d*≈22.5 Å), and the backbone peak of acceptors in the IP direction at *q*=0.42 Å^−1^ (*d*≈15.0 Å) also vanished in blend films, meaning that the acceptor tends to arrange and stack alongside the polymer donor in the blend film.

The surface morphology of films were studied by atomic force microscopy (AFM) and transmission electron microscopy (TEM). As shown in the Supporting Information, Figure S25, blend films of PBDB‐T:H1 and PBDB‐T:H2 both exhibited smooth surface with similar low root‐mean‐square (Rq) values of 1.34 nm and 1.55 nm, indicating the good miscibility between donor and acceptor. TEM images of blend films (Supporting Information, Figure S26 show the nanofiber structure with small phase domain, which is beneficial for the charge transport and separation, thus leading to a high *J*_SC_ and FF.

## Conclusion

Two novel acceptors H1 and H2 were synthesized, which along with Y6 were shown to be triplet materials, and the OSCs afforded a highest PCE of 15 %. Steady and transient photoluminescence and absorption spectroscopy showed that these materials possess strong light absorption and long lifetime excitons, while TD‐DFT calculations revealed that the twisted conformation and D‐A structure led to a considerable *k*_ISC_. Magneto‐photocurrent and electron paramagnetic resonance experiments further revealed the triplet nature of these acceptors. The triplet exciton pairs were generated and split in the blend films to contribute to the PCE, supported by magneto‐photocurrent, transient spectroscopy, EQE, and EL spectroscopy. This work sheds light on understanding the working mechanism of triplet excitons in OSCs.

## Conflict of interest

The authors declare no conflict of interest.

## Supporting information

As a service to our authors and readers, this journal provides supporting information supplied by the authors. Such materials are peer reviewed and may be re‐organized for online delivery, but are not copy‐edited or typeset. Technical support issues arising from supporting information (other than missing files) should be addressed to the authors.

Supplementary

## References

[1a] G. Zhang , J. Zhao , P. C. Y. Chow , K. Jiang , J. Zhang , Z. Zhu , J. Zhang , F. Huang , H. Yan , Chem. Rev. 2018, 118, 3447–3507; 29557657 10.1021/acs.chemrev.7b00535

[1b] C. Yan , S. Barlow , Z. Wang , H. Yan , A. K. Y. Jen , S. R. Marder , X. Zhan , Nat. Rev. Mater. 2018, 3, 18003;

[1c] A. Wadsworth , M. Moser , A. Marks , M. S. Little , N. Gasparini , C. J. Brabec , D. Baran , I. McCulloch , Chem. Soc. Rev. 2019, 48, 1596–1625; 29697109 10.1039/c7cs00892a

[1d] J. Hou , O. Inganäs , R. H. Friend , F. Gao , Nat. Mater. 2018, 17, 119–128. 29358765 10.1038/nmat5063

[2a] J. Xiong , K. Jin , Y. Jiang , J. Qin , T. Wang , J. Liu , Q. Liu , H. Peng , X. Li , A. Sun , X. Meng , L. Zhang , L. Liu , W. Li , Z. Fang , X. Jia , Z. Xiao , Y. Feng , X. Zhang , K. Sun , S. Yang , S. Shi , L. Ding , Sci. Bull. 2019, 64, 1573–1576; 10.1016/j.scib.2019.10.002 36659568

[2b] L. X. Meng , Y. M. Zhang , X. J. Wan , C. X. Li , X. Zhang , Y. B. Wang , X. Ke , Z. Xiao , L. M. Ding , R. X. Xia , H. L. Yip , Y. Cao , Y. S. Chen , Science 2018, 361, 1094–1098; 30093603 10.1126/science.aat2612

[2c] K. Jiang , Q. Wei , J. Y. L. Lai , Z. Peng , H. K. Kim , J. Yuan , L. Ye , H. Ade , Y. Zou , H. Yan , Joule 2019, 3, 3020–3033.

[3a] M. K. Etherington , J. Wang , P. C. Y. Chow , N. C. Greenham , Appl. Phys. Lett. 2014, 104, 063304;

[3b] D. Veldman , S. C. J. Meskers , R. A. J. Janssen , Adv. Funct. Mater. 2009, 19, 1939–1948.

[4] S. Xu , Y. Yuan , X. Cai , C. J. Zhang , F. Hu , J. Liang , G. Zhang , D. Zhang , B. Liu , Chem. Sci. 2015, 6, 5824–5830. 28791088 10.1039/c5sc01733e PMC5520955

[5] Y. Shao , Y. Yang , Adv. Mater. 2005, 17, 2841–2844.

[6] M. Qian , R. Zhang , J. Hao , W. Zhang , Q. Zhang , J. Wang , Y. Tao , S. Chen , J. Fang , W. Huang , Adv. Mater. 2015, 27, 3546–3552. 25946623 10.1002/adma.201500730

[7] L. Yang , W. Gu , L. Lv , Y. Chen , Y. Yang , P. Ye , J. Wu , L. Hong , A. Peng , H. Huang , Angew. Chem. Int. Ed. 2018, 57, 1096–1102; 10.1002/anie.201712011 29215780

[8] S. Liu , H. Zhang , Y. Li , J. Liu , L. Du , M. Chen , R. T. K. Kwok , J. W. Y. Lam , D. L. Phillips , B. Z. Tang , Angew. Chem. Int. Ed. 2018, 57, 15189–15193; 10.1002/anie.201810326 30253012

[9a] H. Uoyama , K. Goushi , K. Shizu , H. Nomura , C. Adachi , Nature 2012, 492, 234–238; 23235877 10.1038/nature11687

[9b] M. J. Leitl , V. A. Krylova , P. I. Djurovich , M. E. Thompson , H. Yersin , J. Am. Chem. Soc. 2014, 136, 16032–16038. 25260042 10.1021/ja508155x

[10a] J. Yuan , Y. Zhang , L. Zhou , G. Zhang , H.-L. Yip , T.-K. Lau , X. Lu , C. Zhu , H. Peng , P. A. Johnson , M. Leclerc , Y. Cao , J. Ulanski , Y. Li , Y. Zou , Joule 2019, 3, 1140–1151;

[10b] T. Yan , W. Song , J. Huang , R. Peng , L. Huang , Z. Ge , Adv. Mater. 2019, 31, 1902210; 10.1002/adma.201902210 31411359

[10c] Y. Cui , H. Yao , J. Zhang , T. Zhang , Y. Wang , L. Hong , K. Xian , B. Xu , S. Zhang , J. Peng , Z. Wei , F. Gao , J. Hou , Nat. Commun. 2019, 10, 2515. 31175276 10.1038/s41467-019-10351-5 PMC6555805

[11] L. Yang , L. Qin , Y. Xu , H. Zhang , L. Lv , K. Chen , X. Sui , Y. Zhong , Y. Guo , F. Gao , J. Zhao , Y. Li , X. Liu , Y. Yi , X. Lu , A. Peng , H. Huang , Sci. China Chem. 2019, 62, 897–903.

[12] R. Wang , J. Yuan , R. Wang , G. Han , T. Huang , W. Huang , J. Xue , H. C. Wang , C. Zhang , C. Zhu , P. Cheng , D. Meng , Y. Yi , K. H. Wei , Y. Zou , Y. Yang , Adv. Mater. 2019, 31, 1904215. 10.1002/adma.201904215 31495980

[13] C. S. Ponseca, Jr. , P. Chabera , J. Uhlig , P. Persson , V. Sundstrom , Chem. Rev. 2017, 117, 10940–11024. 28805062 10.1021/acs.chemrev.6b00807

[14a] K. Wang , C. J. Zheng , W. Liu , K. Liang , Y. Z. Shi , S. L. Tao , C. S. Lee , X. M. Ou , X. H. Zhang , Adv. Mater. 2017, 29, 1701476; 10.1002/adma.201701476 29116652

[14b] T. Hu , G. Han , Z. Tu , R. Duan , Y. Yi , J. Phys. Chem. C 2018, 122, 27191–27197.

[15] W. Yang , J. Zhao , C. Sonn , D. Escudero , A. Karatay , H. G. Yaglioglu , B. Küçüköz , M. Hayvali , C. Li , D. Jacquemin , J. Phys. Chem. C 2016, 120, 10162–10175.

[16] V. Ern , R. E. Merrifield , Phys. Rev. Lett. 1968, 21, 609–611.

[17] B. Hu , L. Yan , M. Shao , Adv. Mater. 2009, 21, 1500–1516.

[18a] Z. Xu , B. Hu , Adv. Funct. Mater. 2008, 18, 2611–2617;

[18b] D. Beljonne , Z. Shuai , G. Pourtois , J. Bredas , J. Phys. Chem. A 2001, 105, 3899–3907.

[19a] J. Kalinowski , J. Szmytkowski , W. Stampor , Chem. Phys. Lett. 2003, 378, 380–387;

[19b] H. D. Burrows , M. Fernandes , J. Seixas de Melo , A. P. Monkman , S. Navaratnam , J. Am. Chem. Soc. 2003, 125, 15310–15311; 14664573 10.1021/ja037254f

[19c] B. Brocklehurst , Nature 1969, 221, 921–923.

[20] L. Wang , H. Yu , R. S. Ullah , M. Haroon , S. Fahad , J. Li , T. Elshaarani , R. U. Khan , A. Nazir , Polym. Chem. 2018, 9, 3306–3335. 10.1039/c7tb03332j 32254311

[21] S. Mao , N. Hirota , Mol. Phys. 1974, 27, 309–326.

[22] F. Kraffert , R. Steyrleuthner , S. Albrecht , D. Neher , M. C. Scharber , R. Bittl , J. Behrends , J. Phys. Chem. C 2014, 118, 28482–28493.

[23] M. Luo , C. Zhao , J. Yuan , J. Hai , F. Cai , Y. Hu , H. Peng , Y. Bai , Z. Tan , Y. Zou , Mater. Chem. Front. 2019, 3, 2483–2490.

[24a] H. Chen , Z. Hu , H. Wang , L. Liu , P. Chao , J. Qu , W. Chen , A. Liu , F. He , Joule 2018, 2, 1623–1634;

[24b] X. Zhong , H. Chen , M. Wang , S. Gan , Q. He , W. Chen , F. He , Macromolecules 2019, 52, 2393–2401.

[25] F. Zhao , K. Wang , J. Duan , X. Zhu , K. Lu , C. Zhao , C. Zhang , H. Yu , B. Hu , Sol. RRL 2019, 3, 1900063.

[26] X. Zhu , K. Wang , J. He , L. Zhang , H. Yu , D. He , B. Hu , J. Phys. Chem. C 2019, 123, 20691–20697.

[27a] P. C. Chow , S. Gelinas , A. Rao , R. H. Friend , J. Am. Chem. Soc. 2014, 136, 3424–3429; 24521399 10.1021/ja410092n

[27b] A. Rao , P. C. Chow , S. Gelinas , C. W. Schlenker , C. Z. Li , H. L. Yip , A. K. Jen , D. S. Ginger , R. H. Friend , Nature 2013, 500, 435–439; 23925118 10.1038/nature12339

[27c] L. Xue , Y. Yang , J. Xu , C. Zhang , H. Bin , Z. Zhang , B. Qiu , X. Li , C. Sun , L. Gao , J. Yao , X. Chen , Y. Yang , M. Xiao , Y. Li , Adv. Mater. 2017, 29, 1703344. 10.1002/adma.201703344 28859234

[28] J. Liu , S. Chen , D. Qian , B. Gautam , G. Yang , J. Zhao , J. Bergqvist , F. Zhang , W. Ma , H. Ade , O. Inganäs , K. Gundogdu , F. Gao , H. Yan , Nat. Energy 2016, 1, 16089.

[29] J. Yuan , T. Huang , P. Cheng , Y. Zou , H. Zhang , J. L. Yang , S. Y. Chang , Z. Zhang , W. Huang , R. Wang , D. Meng , F. Gao , Y. Yang , Nat. Commun. 2019, 10, 570. 30718494 10.1038/s41467-019-08386-9 PMC6362024

[30] D. Qian , Z. Zheng , H. Yao , W. Tress , T. R. Hopper , S. Chen , S. Li , J. Liu , S. Chen , J. Zhang , X. K. Liu , B. Gao , L. Ouyang , Y. Jin , G. Pozina , I. A. Buyanova , W. M. Chen , O. Inganas , V. Coropceanu , J. L. Bredas , H. Yan , J. Hou , F. Zhang , A. A. Bakulin , F. Gao , Nat. Mater. 2018, 17, 703–709. 30013057 10.1038/s41563-018-0128-z

[31] Y. Wang , D. Qian , Y. Cui , H. Zhang , J. Hou , K. Vandewal , T. Kirchartz , F. Gao , Adv. Energy Mater. 2018, 8, 1801352.

[32] J. Yao , T. Kirchartz , M. S. Vezie , M. A. Faist , W. Gong , Z. He , H. Wu , J. Troughton , T. Watson , D. Bryant , J. Nelson , Phys. Rev. Appl. 2015, 4, 014020.

[33] K. Vandewal , S. Albrecht , E. T. Hoke , K. R. Graham , J. Widmer , J. D. Douglas , M. Schubert , W. R. Mateker , J. T. Bloking , G. F. Burkhard , A. Sellinger , J. M. Frechet , A. Amassian , M. K. Riede , M. D. McGehee , D. Neher , A. Salleo , Nat. Mater. 2014, 13, 63–68. 24240240 10.1038/nmat3807

[34] W. Zhao , T. S. Cheung , N. Jiang , W. Huang , J. W. Y. Lam , X. Zhang , Z. He , B. Z. Tang , Nat. Commun. 2019, 10, 1595. 30962451 10.1038/s41467-019-09561-8 PMC6453937

[35] A. P. Monkman , H. D. Burrows , L. J. Hartwell , L. E. Horsburgh , I. Hamblett , S. Navaratnam , Phys. Rev. Lett. 2001, 86, 1358–1361. 11178083 10.1103/PhysRevLett.86.1358

[36] K. Vandewal , J. Benduhn , V. Nikolis , Sustainable Energy Fuels 2018, 2, 538–544.

