# Supplementary material for: Triplet Acceptors with a D‐A Structure and Twisted Conformation for Efficient Organic Solar Cells
Source: Angew Chem Int Ed Engl. 2020 Jun 9;59(35):15043–9. doi: 10.1002/anie.202006081 (PMC7497160; doi:10.1002/anie.202006081)
Supplement: Supplementary file 1 — Supplementary [file ANIE-59-15043-s001.pdf]

Angewandte Chemie
Eine Zeitschrift der Gesellschaft Deutscher Chemiker

Supporting Information

Triplet Acceptors with a D-A Structure and Twisted Conformation for Efficient Organic Solar Cells

Linqing Qin+, Xingzheng Liu+, Xin Zhang+, Jianwei Yu, Lei Yang, Fenggui Zhao, Miaofei Huang, Kangwei Wang, Xiaoxi Wu, Yuhao Li, Hao Chen, Kai Wang, Jianlong Xia, Xinhui Lu, Feng Gao, Yuanping Yi,* and Hui Huang**

anie_202006081_sm_miscellaneous_information.pdf

Supporting Information
©Wiley-VCH 2019
69451 Weinheim, Germany

Triplet acceptors with D-A structure and twisted conformation for efficient organic solar cells

Linqing Qin, Xingzheng Liu, Xin Zhang, Jianwei Yu, Lei Yang, Fenggui Zhao, Miaofei Huang, Kangwei Wang, Xiaoxi Wu, Yuhao Li, Hao Chen, Kai Wang, Jianlong Xia, Xinhui Lu, Feng Gao*, Yuanping Yi*, and Hui Huang*.

Abstract: Triplet acceptors have been developed to construct high performance organic solar cells (OSCs) since the long lifetime and diffusion range of triplet excitons may dissociate into free charges instead of net recombination when the energy levels of triplet states (T_1) are close to those of charge transfer states (^{3}CT). The current triplet acceptors were designed by introducing heavy atoms to enhance the intersystem crossing, limiting their applications. Herein, two twisted acceptors without heavy atoms, analogues of Y6, were constructed with large π-conjugated core and D-A structure, proved to be triplet materials, leading to a high performance OSCs. The mechanism of triplet excitons were investigated to show that the twisted and D-A structures result in large spin orbital coupling (SOC) and small energy gap between the singlet and triplet states, and thus efficient intersystem crossing. Moreover, the energy level of T_1 is close to ^{3}CT, facilitating the split of triplet exciton to free charges. This contribution provided a novel road to high performance triplet acceptors.

DOI: 10.1002/anie.2020XXXXX

Experimental Procedures

Materials

PBDB-T, PM6, Y6 and PDINO were purchased from Solarmer Inc. and used directly without purification. Other chemical reagents and solvents were purchased from commercial resources.

Compound **1**, 2-(6-fluoro-3-oxo-2,3-dihydro-1H-inden-1-ylidene)malononitrile (mixture of isomers) and 2-(6-chloro-3-oxo-2,3-dihydro-1H-inden-1-ylidene)malononitrile (mixture of isomers) were purchased from Hyper Inc.

Synthesis of H1

To a solution of compound **1** (154.1 mg, 0.150 mmol), 2-(6-fluoro-3-oxo-2,3-dihydro-1H-inden-1-ylidene)malononitrile (mixture of isomers) (159.1 mg, 0.750 mmol) and pyridine (0.6 mL) in $CHCl_3$ (50 mL) was heated at 60 °C overnight. After cooling to room temperature, the mixture was concentrated to 5 mL and poured into methanol (50 mL) and then filtered. The residue was purified via column chromatography on silica gel using chloroform as the eluent to afford compound **H1** (191.4 mg, 0.135 mmol, yield = 90.1%) as a dark blue solid with metallic luster. (Note: there exists three isomers in the **H1** resulting from the mixture of two isomers in the end group, thus the final product cannot be separated due to their rather similar chemical structures.) ^{1}H NMR (400MHz, $CDCl_3$, δ): 9.17–9.15 (m, 2H), 8.76–8.71 (m, 0.21H), 8.43–8.37 (m, 1.79H), 7.98–7.93 (m, 1.79H), 7.62–7.56 (m, 0.21H), 7.45–7.40 (m, 2H), 4.85–4.74

(m, 4H), 3.26–3.20 (m, 4H), 2.17–2.06 (m, 2H), 1.93–1.85 (m, 4H), 1.53–1.45 (m, 4H), 1.37–1.19 (m, 32H), 1.11–0.98 (m, 12H), 0.89–0.84 (m, 6H), 0.79–0.73 (m, 6H), 0.69–0.64 (m, 6H); ^{13}C NMR (100MHz, $CDCl_3$, δ): 187.12, 180.10, 166.20, 165.50, 153.54, 147.58, 145.11, 142.42, 140.15, 137.91, 135.83, 135.26, 133.82, 133.31, 133.31, 130.33, 127.41, 121.68, 121.45, 121.45, 120.75, 115.14, 115.14, 114.56, 114.56, 113.65, 113.65, 112.66, 110.56, 100.01, 77.38, 69.07, 55.78, 40.49, 31.96, 31.21, 29.87, 29.82, 29.67, 29.56, 29.51, 29.38, 27.75, 23.36, 22.88, 14.13, 13.75, 10.33, 10.33, 10.27. ; HR-MS (MALDI-TOF): *m/z* calcd. for $C_{82}H_{88}F_2N_8O_2S_5$: 1414.5602; found: 1414.5 $(M+H)^+$. Elem. Anal. Calcd for $C_{82}H_{88}F_2N_8O_2S_5$: C, 69.68; N, 7.74; H, 6.68. Found: C, 70.72; N, 7.36; H, 6.76.

Synthesis of H2

To a solution of compound **1** (154.1 mg, 0.150 mmol), 2-(6-chloro-3-oxo-2,3-dihydro-1H-inden-1-ylidene)malononitrile (mixture of isomers) (171.5 mg, 0.750 mmol) and pyridine (0.6 mL) in $CHCl_3$ (50 mL) was heated at 60 °C overnight. After cooling to room temperature, the mixture was concentrated to 5 mL and poured into methanol (50 mL) and then filtered. The residue was purified via column chromatography on silica gel using chloroform as the eluent to afford compound **H2** (191.9 mg, 0.132 mmol, yield = 88.3%) as a dark blue solid. (Note: there exists three isomers in the **H2** resulting from the mixture of two isomers in the end group, thus the final product cannot be separated due to their rather similar chemical structures.) ^1H NMR (400MHz, $CDCl_3$, δ): 9.20–9.17 (m, 2H), 8.70–8.63 (m, 2H), 7.90–7.87 (m, 2H), 7.70–7.69 (m, 2H), 4.84–4.72 (m, 4H), 3.29–3.20 (m, 4H), 2.16–2.06 (m, 2H), 1.92–1.85 (m, 4H), 1.52–1.44 (m, 4H), 1.36–1.21 (m, 32H), 1.11–0.98 (m, 12H), 0.88–0.84 (m, 6H), 0.79–0.73 (m, 6H), 0.68–0.64 (m, 6H); ^{13}C NMR data of **H2** cannot be obtained due to the poor solubility in chloroform; HR-MS (MALDI-TOF): *m/z* calcd. for $C_{82}H_{88}Cl_2N_8O_2S_5$: 1446.5011; found: 1445.4 $(M+H)^+$. Elem. Anal. Calcd for $C_{82}H_{88}F_2N_8O_2S_5$: C, 68.13; N, 7.57; H, 6.53. Found: C, 67.89; N, 7.77; H, 6.26.

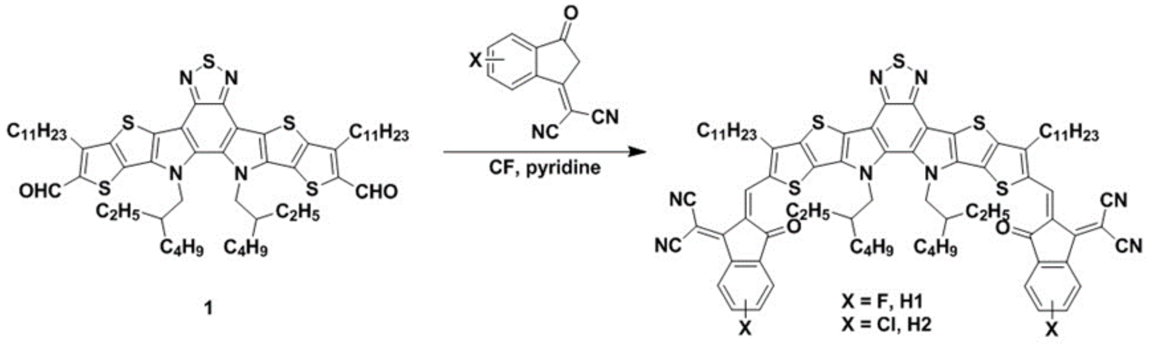

Scheme S1. Synthetic Route to H1 and H2.

Characterization.

^1H NMR and ^{13}C NMR spectrum were recorded on a Bruker AVANCE 400 MHz NMR spectrometer. MALDI-TOF spectrum were obtained from a Bruker Autoflex III Mass Spectrometer. Elemental analysis was performed on a FLASH EA 1112 elemental analyzer. The UV-vis absorption spectra of chloroform solution and thin film were measured on a Gary 60 UV-vis Spectrophotometer and the thin film was spin coated on a quartz substrate. The electrochemical cyclic voltammetry (CV) measurements were carried out under nitrogen in 0.1 M tetrabutylammonium hexafluorophosphate (Bu4NPF6) acetonitrile solution at a potential scan speed at 0.05 V/s. The ferrocene/ferrocenium (Fc/Fc+) was used as external standard in the measurement. A glassy-carbon disc coated with donor or acceptor film was used as the working electrode, a Pt wire was used as the counter electrode and an Ag/AgCl electrode was used as a reference electrode. AFM images were obtained by Bruker Dimension Icon in the tapping mode. All the samples were spin casted on silicon substrates. TEM images were performed by HT7700Ex instrument at 110 kV accelerating voltage. Time-Dependent Density functional theory (TD-DFT) calculations were performed on the B3LYP/6-31G(d, p) level by Gaussian16 program. GIWAXS measurements were carried out with a Xeuss 2.0 SAXS/WAXS laboratory beamline using a Cu X-ray source (8.05 keV, 1.54 Å) and a Pilatus3R 300K detector, and samples were prepared on Si substrates. The incidence angle is 0.2°. Transient photoluminescence tests were carried out on a FLS920 Cary Eclipse Fluorescence Spectrophotometer. The magneto-photocurrent (MPC) were measured by a PR650 spectroradiometer. A Keithley2612 sourcemeter was used to provide a laser pulse, and channel A also recorded the current intensity. The measurements were carried out at room temperature under ambient condition. Transient absorption spectroscopy of the films were performed on a commercial nanosecond transient absorption spectrometer (EOS, Ultrafast Systems). 1kHz laser pulses at 1040 nm with <400 fs duration were generated by a 200 kHz amplified laser system (Spirit 1040-8-SHG, Newport Corporation). The 1040nm beam was directed into a TOPAS optical parametric amplifier to generate pump pulse. The probe beam was a white light continuum beam spanning 360–900 nm spectral region. The excitation fluence in each measurement was around 36 μJ. Transient absorption spectroscopy of solutions were performed on Edinburgh LP980. EPR measurements were performed on Bruker A300-10/12, and the samples were irradiated with a mixed light from Xenon lamp with the range of 380-800 nm and 300 W power.

Device fabrication and characterization.

Organic solar cells were fabricated with a conventional structure of ITO/PEDOT:PSS/active layer/PDINO/Al, where the active layer is consisted of donor polymer and acceptors. Patterned indium tin oxide (ITO) glass was cleaned in an ultrasonic bath with detergent, deionized water, acetone and isopropanol, dried in an oven at 60 ºC overnight and treated in an ultraviolet ozone for 25 min. 80 μL PEDOT:PSS solution was then spin-coated onto the cleaned ITO glass (3500 rpm, 35 s), and baked at 150 ºC for 15 min. Blends of

PBDB-T:H1, PBDB-T:H2 and PM6:Y6 of 1:1.2 with 0.5% 1-chloronaphthalene (CN) as additive were fully dissolved in chloroform solution (CF) at a total concentration of 18 mg/mL. Then, the solution was spin-coated on the substrate to form the active layer, and the thin films were annealed at 110℃ for 10 min. Then a thin layer of PDINO was spin-coated on the films. After that the films were transferred into a vacuum evaporator connected to the glove box. Finally, about 100 nm Al were deposited onto the active layer sequentially by thermal evaporation at a pressure under 10-5 Pa. The current density-voltage (J-V) characterizations were recorded under air mass (AM) 1.5G using a Newport solar simulator. Besides, the external quantum efficiency spectrum was measured using Enlitech QE-R system. The bulk charge transport mobility was investigated by the space charge limit current (SCLC) method. The hole mobility were measured with a device structure of ITO/PEDOT:PSS/active layer/MoO_3/Ag, while electron mobility were characterized with a device structure of ITO/ZnO/active layer/PDINO/Al. Electron mobility and hole mobility data were calculated from *J*-*V* curves and fitting the results to SCLC method by using the equation: $J = (9\mu\varepsilon_0\varepsilon_r V^2)/8L^3$. Where μ is the hole or electron mobility, ε_0 is the permittivity of free space, ε_r is the relative permittivity of the material, *V* is the voltage drop across the device and L is the thickness of the film. For Charge-transfer state (CT) characterizations, the E_{CT} can be determined by fitting the tail of EQE or EL spectra of the CT states with the following equations:

$$\mathrm{EQE}(E) = fE\exp\left(-\frac{(E - E_{\mathrm{CT}} - \lambda)^2}{4\lambda kT}\right)$$

$$\mathrm{EL}(E) = fE^3\exp\left(-\frac{(E - E_{\mathrm{CT}} + \lambda)^2}{4\lambda kT}\right)$$

where E is the photon energy, k is the Boltzmann constant, T is the absolute temperature, λ is the reorganization energy and f is a factor proportional.

Results and Discussion

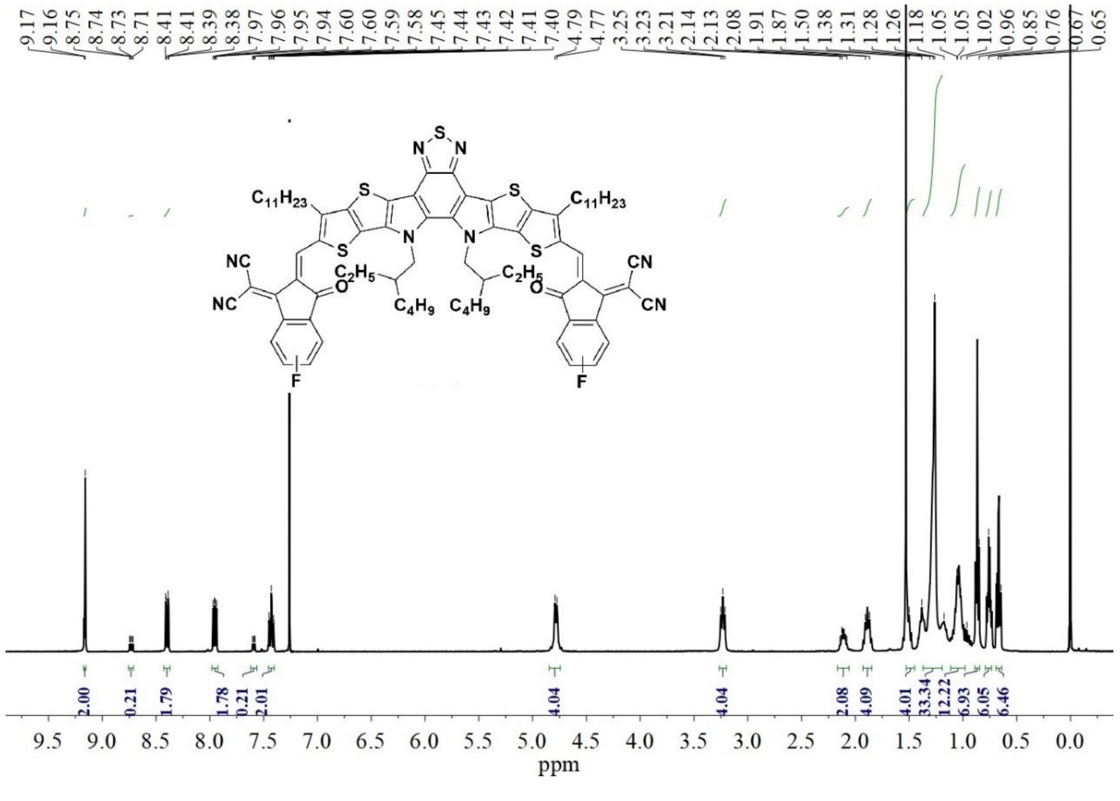

Figure S1. ^{1}H NMR (400 MHz) spectrum of H1 at 298 K.

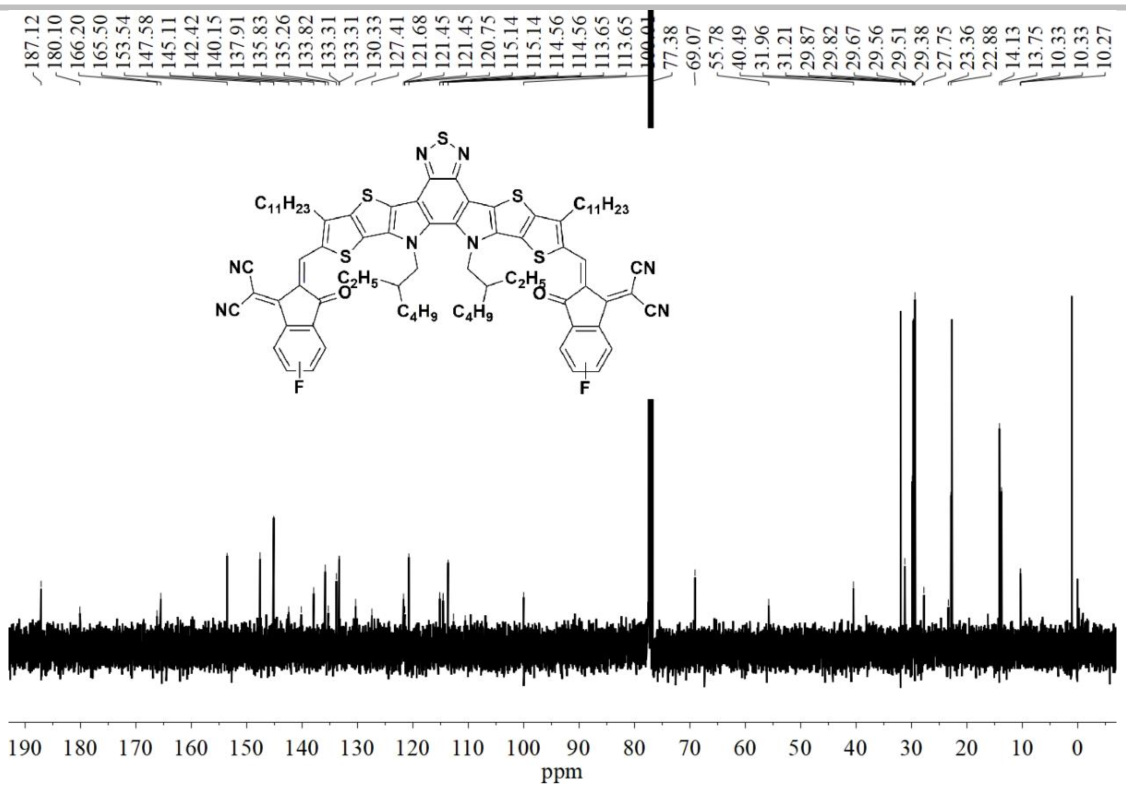

Figure S2. ^{13}C NMR (100 MHz) spectrum of H1 at 298 K.

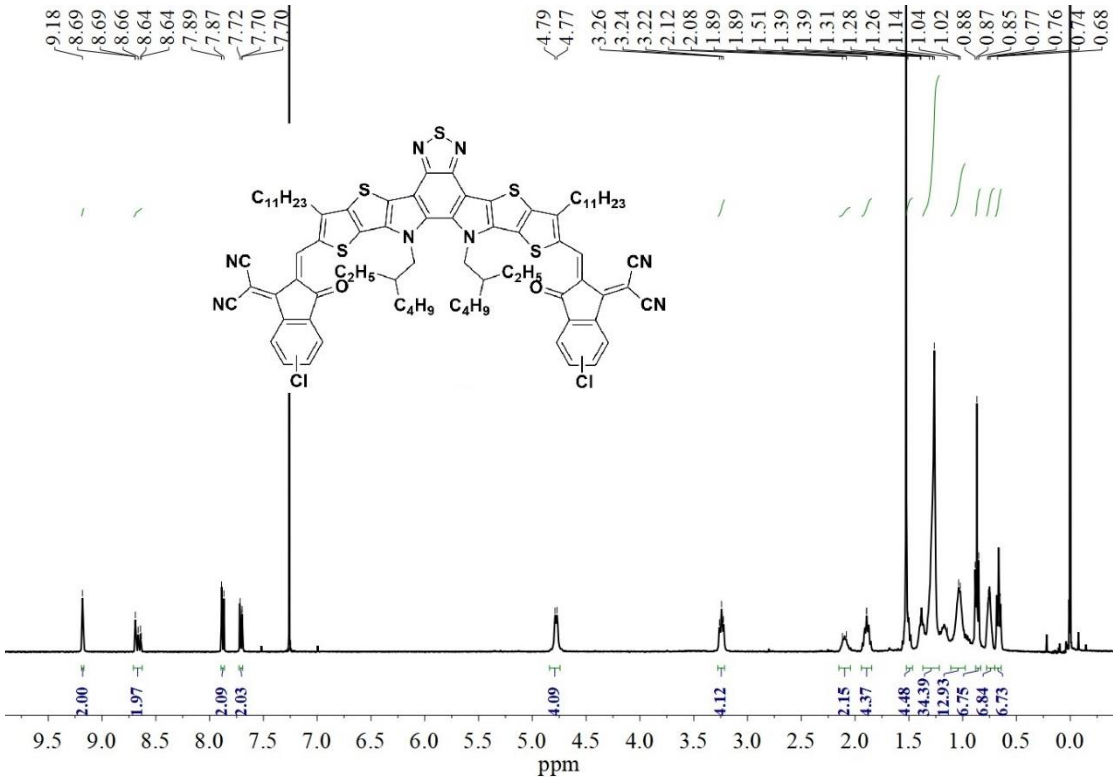

Figure S3. ^{1}H NMR (400 MHz) spectrum of H2 at 298 K.

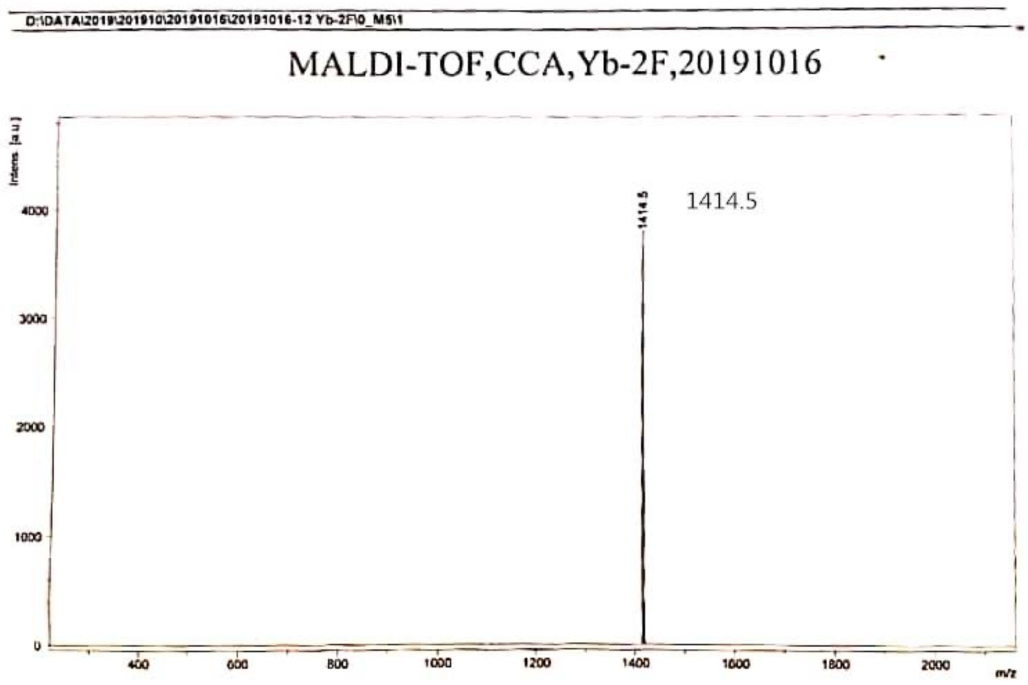

Figure S4. MALDI-TOF spectrum of H1 (sample named Yb-2F)

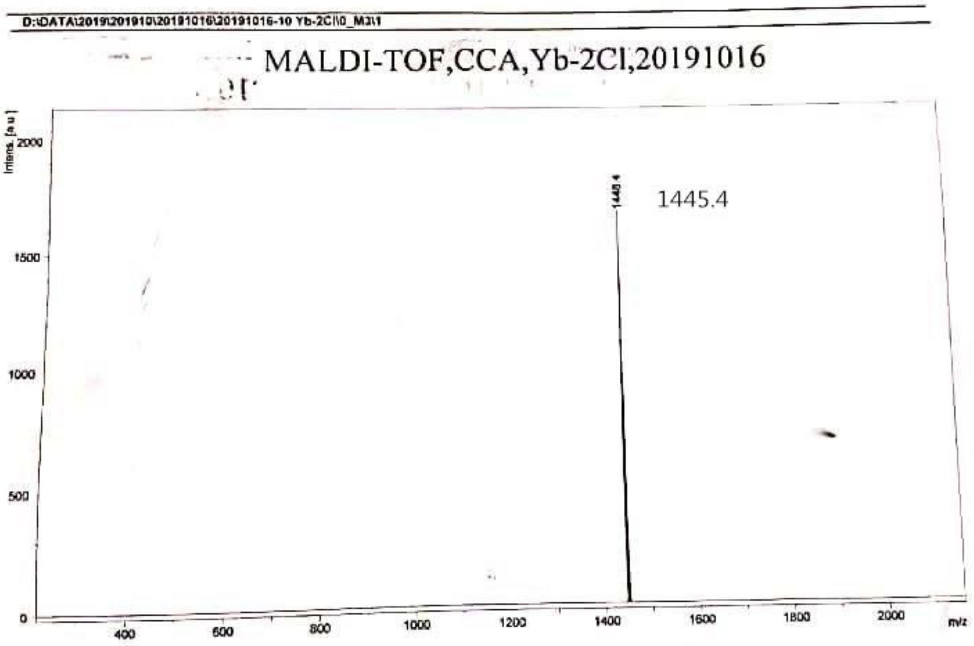

Figure S5. MALDI-TOF spectrum of H2 (sample named Yb-2Cl).

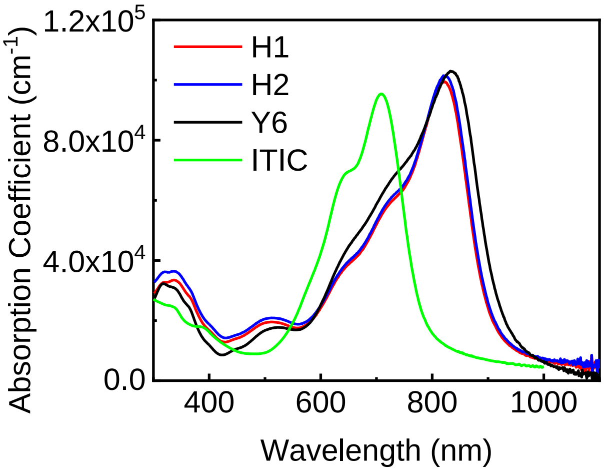

Figure S6. Absorption coefficient of H1, H2, Y6 and ITIC films. The absorption coefficient of ITIC was evaluated to be 9.65×10^4 cm^{-1}.

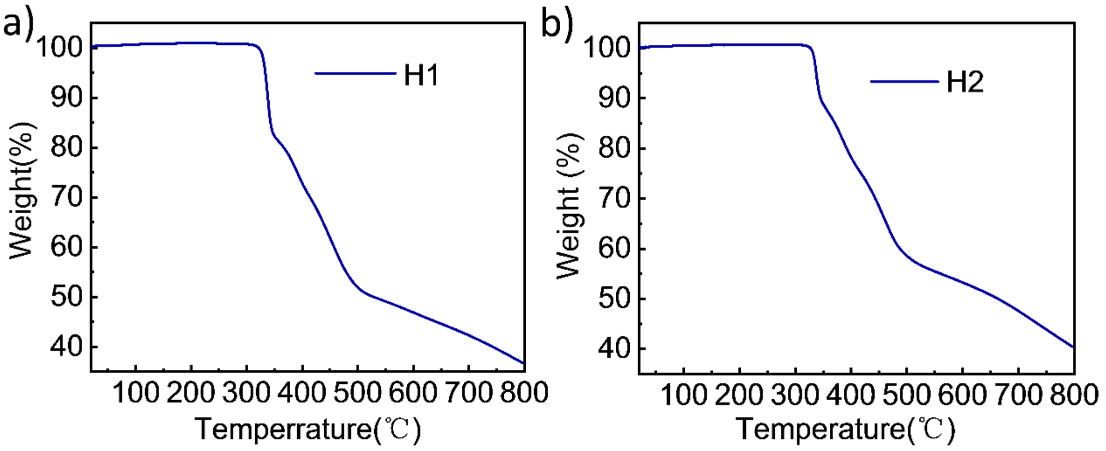

Figure S7. Thermogravimetric analysis curves of a) H1 and b) H2 with heating rate of 20 K min^{-1}

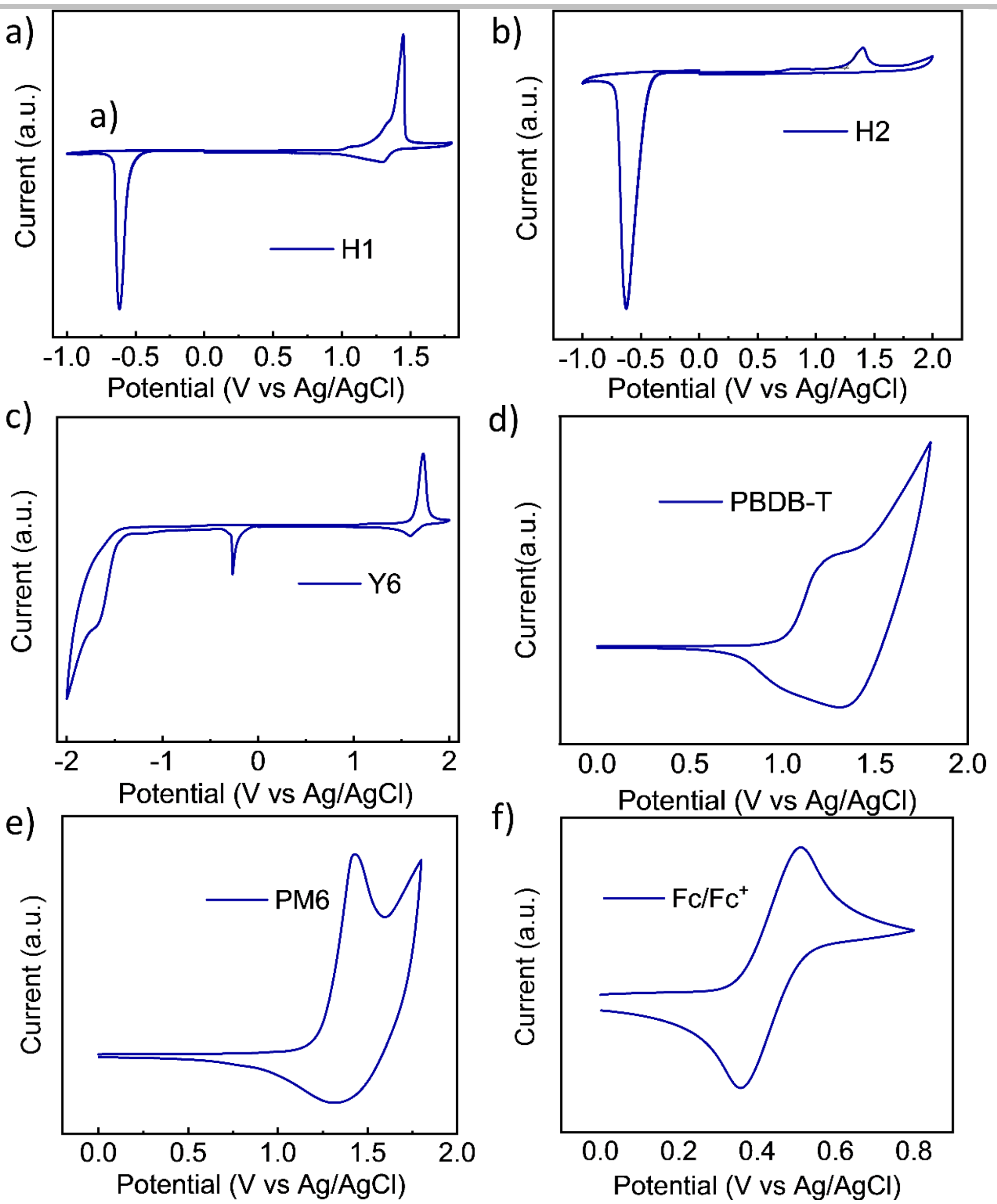

Figure S8. Cyclic voltammetry diagrams of a) H1, b) H2, c) Y6, d) PBDB-T, e) PM6 and f) ferrocene/ferrocenium.

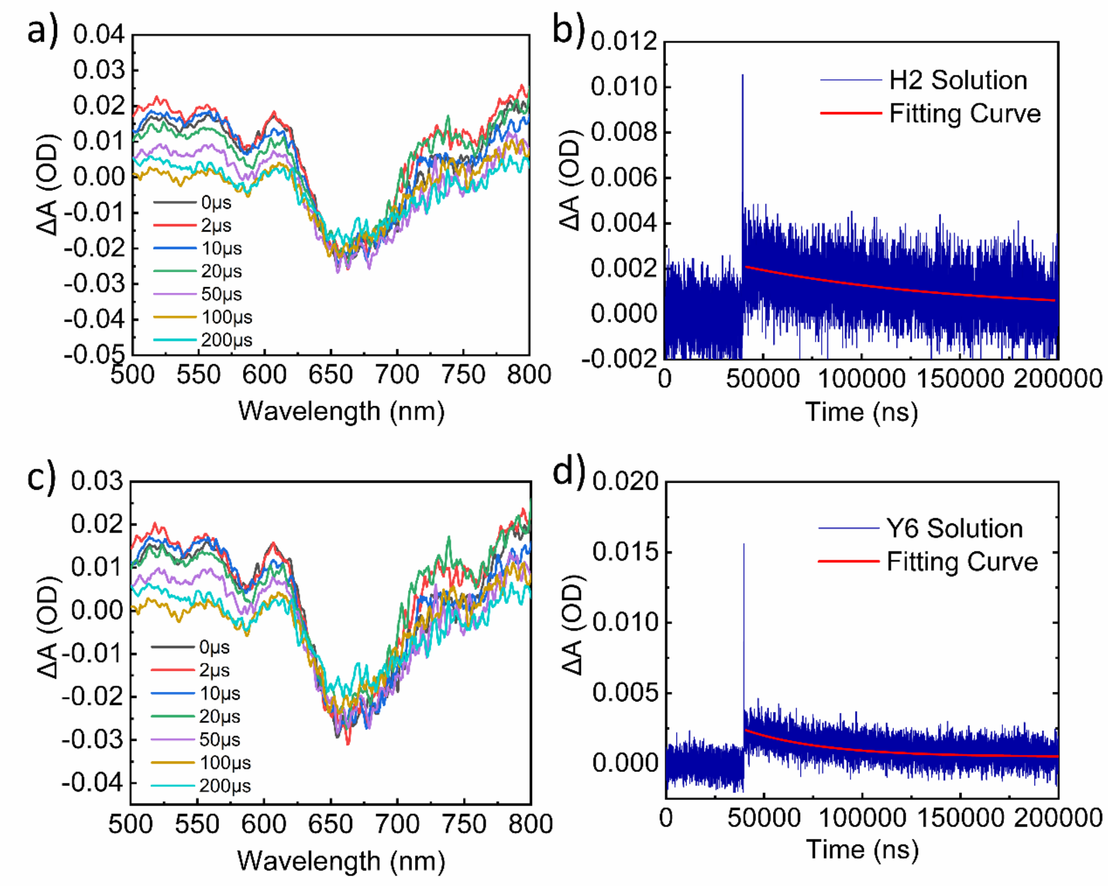

Figure S9. Transient absorption spectra of a) H2 and c) Y6 solutions. Decay traces of b) H2 and d) Y6 probed at 560nm.

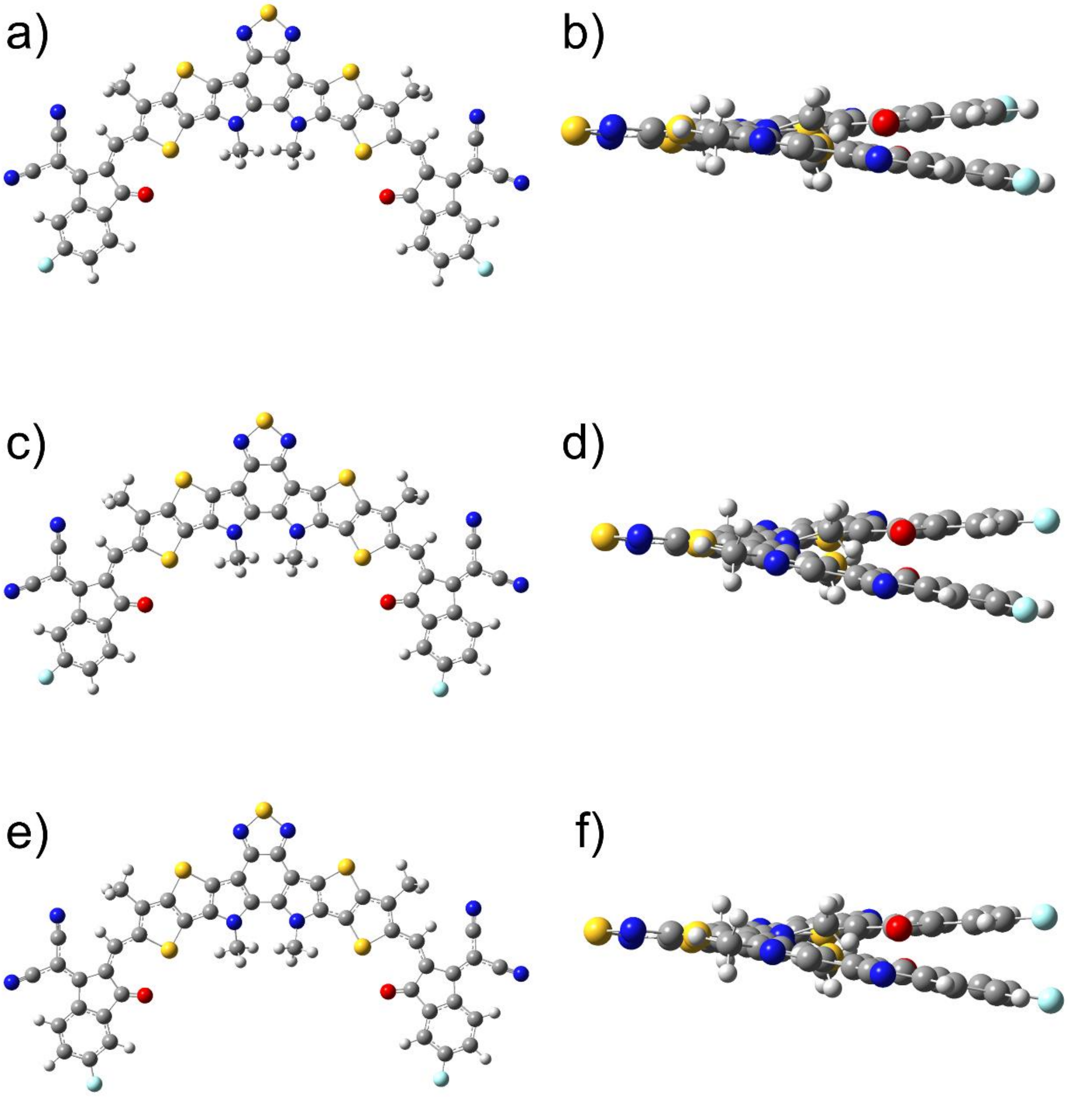

Figure S10. Top view and side view of molecular structure of three isomers of H1 calculated by Gaussian16 program at B3LYP/6-31G(d) level.

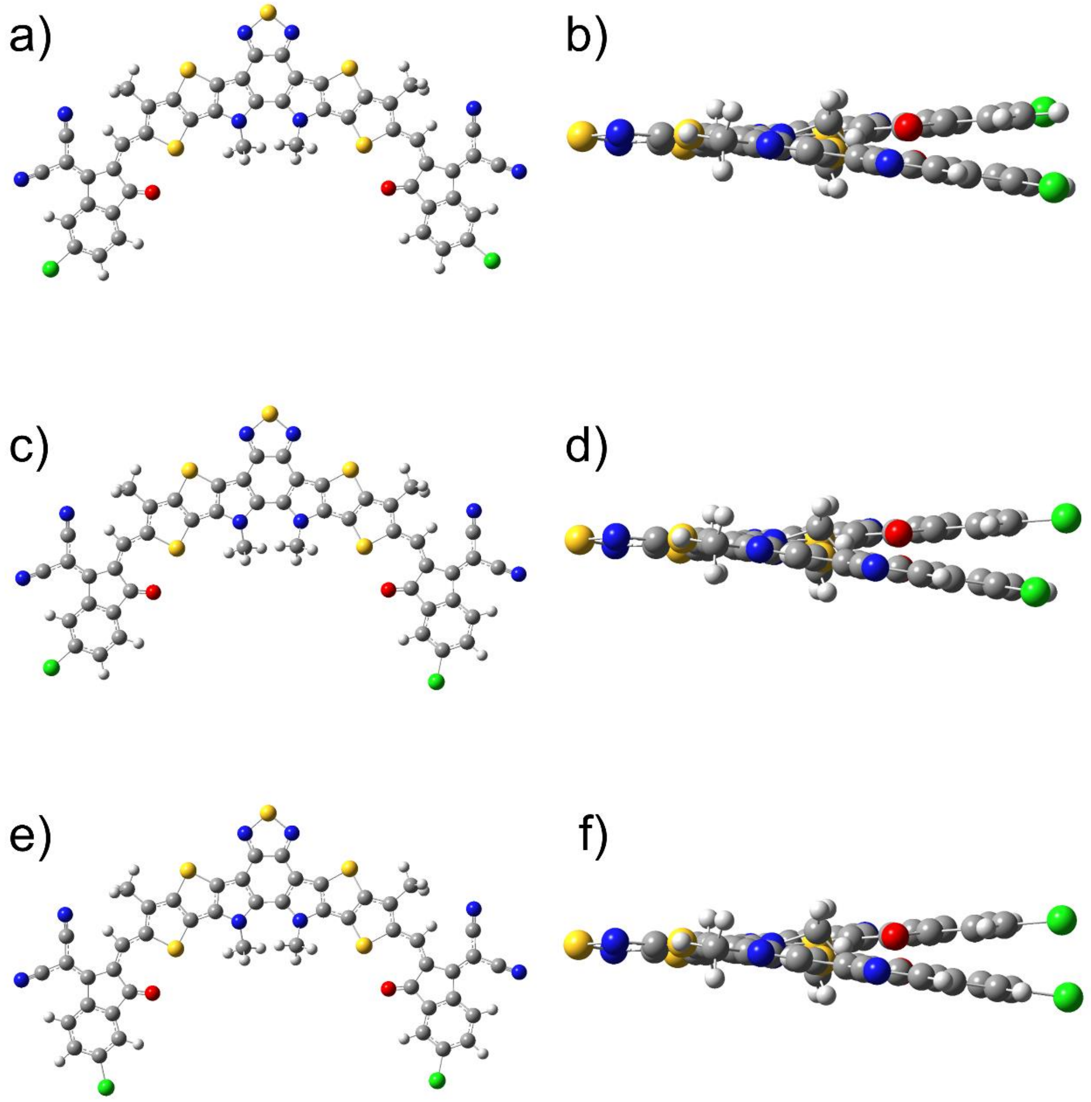

Figure S11. Top view and side view of molecular structure of three isomers of H2 calculated by Gaussian16 program at B3LYP/6-31G(d) level.

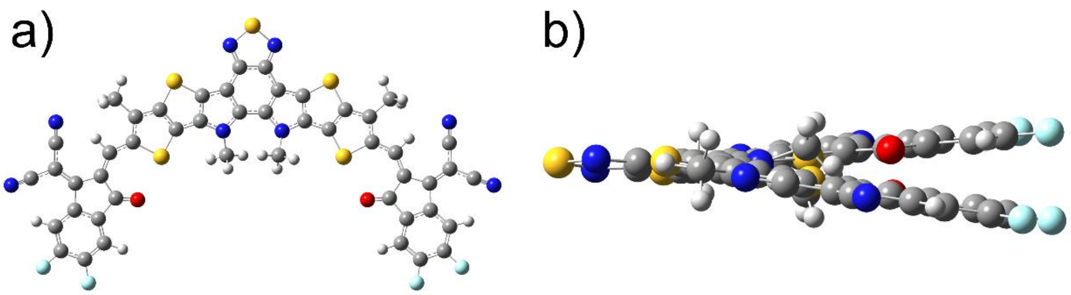

Figure S12. Top view and side view of molecular structure of Y6 calculated by Gaussian16 program at B3LYP/6-31G(d) level.

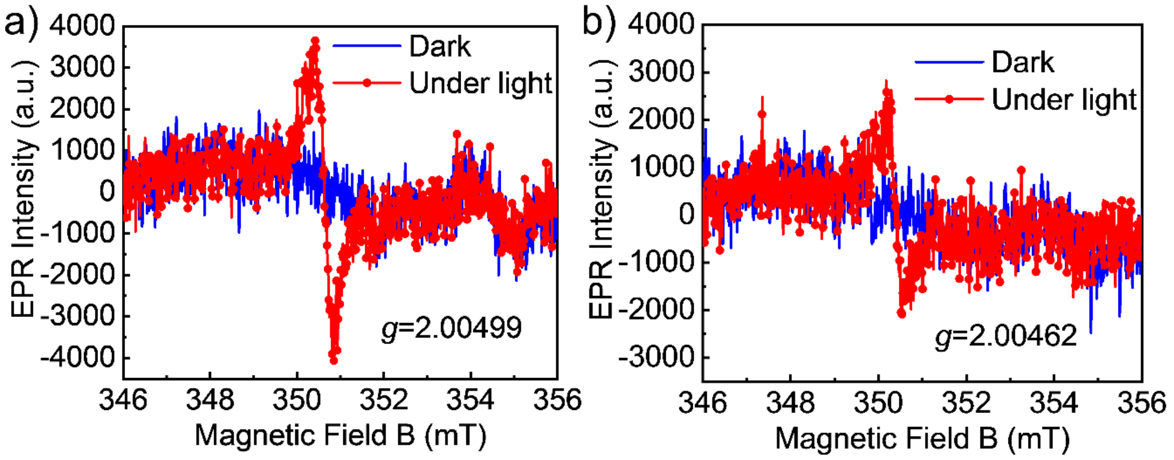

Figure S13. EPR spectra of a) H2 and b) Y6 powder samples in dark and under light (a mixed light from Xenon lamp with the range of 380-800 nm and 300 W power) conditions.

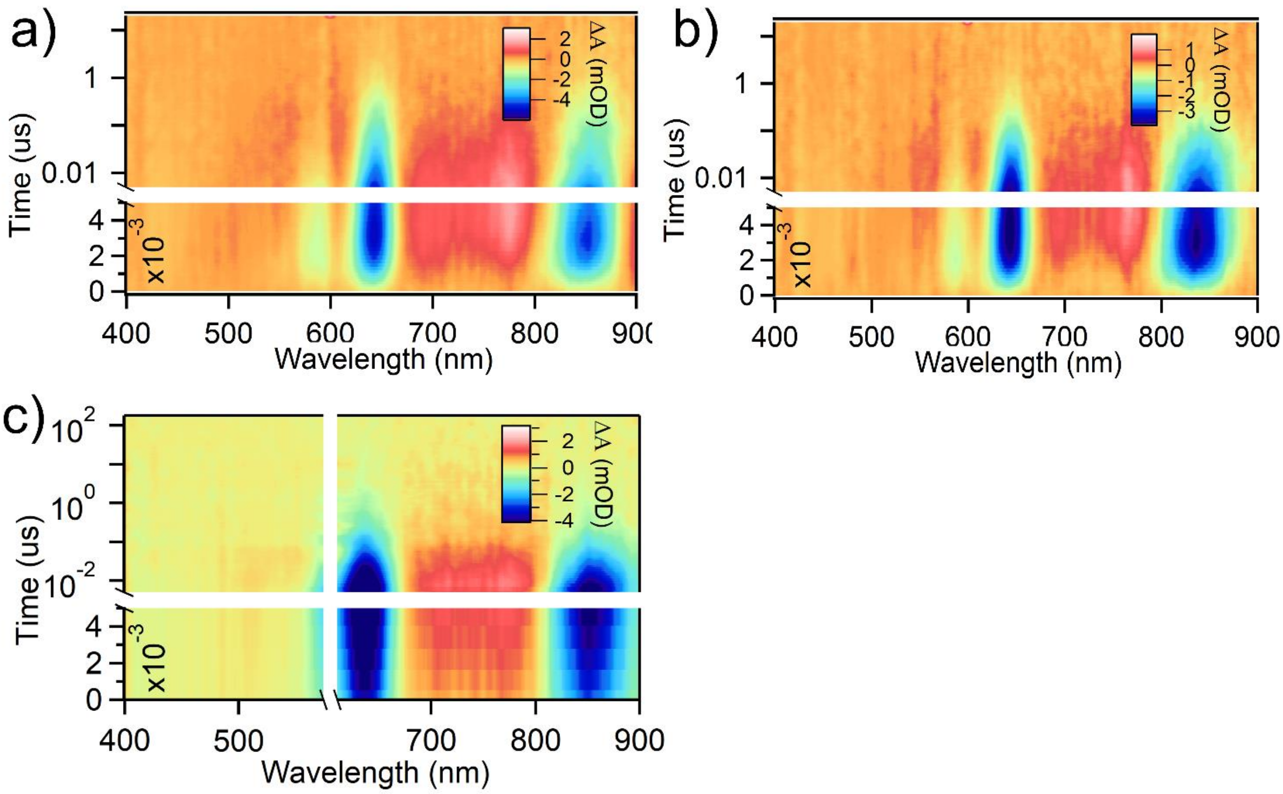

Figure S14. Transient absorption 2D spectra of a) PBDB-T:H1, b) PBDB-T:H2 and c) PM6:Y6 blend film.

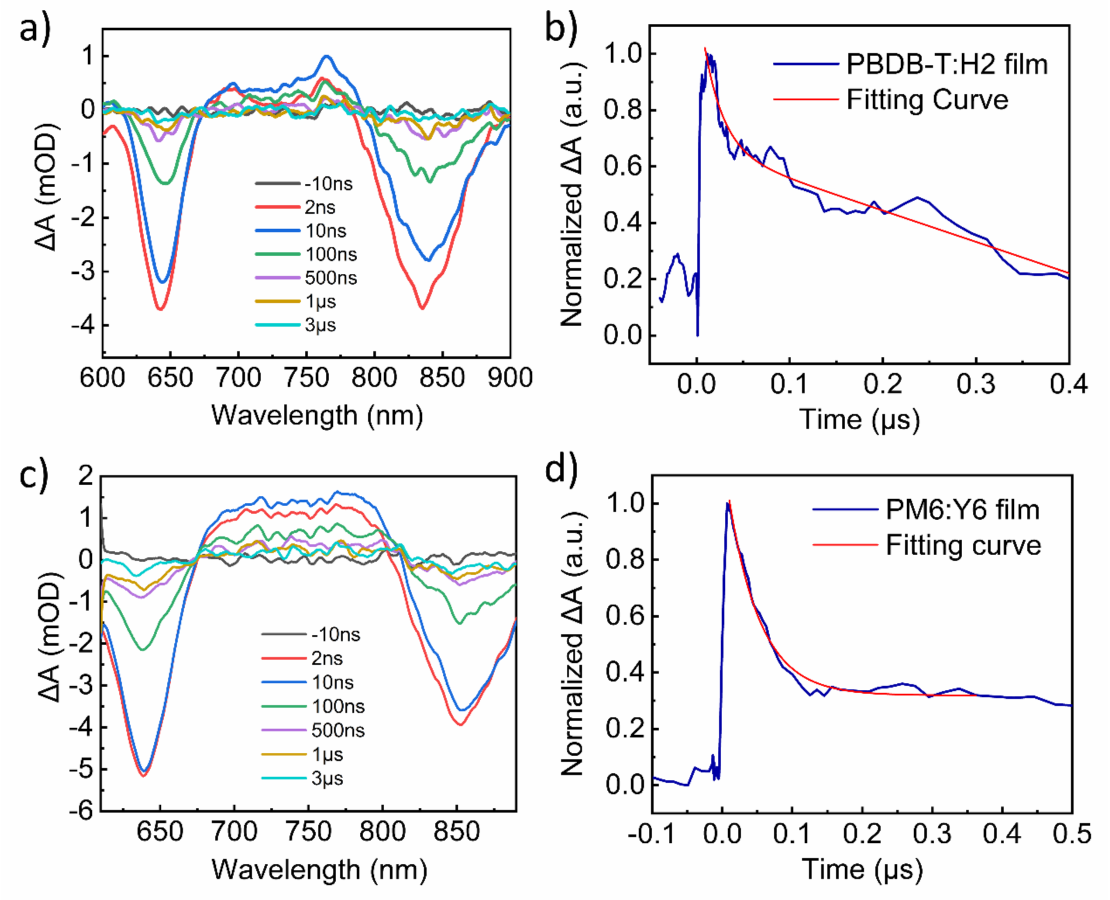

Figure S15. Transient absorption spectra of a) PBDB-T:H2 and c) PM6:Y6 blend film. Decay traces of b) PBDB-T:H2 and d) PM6:Y6 blend film probed at 770 nm.

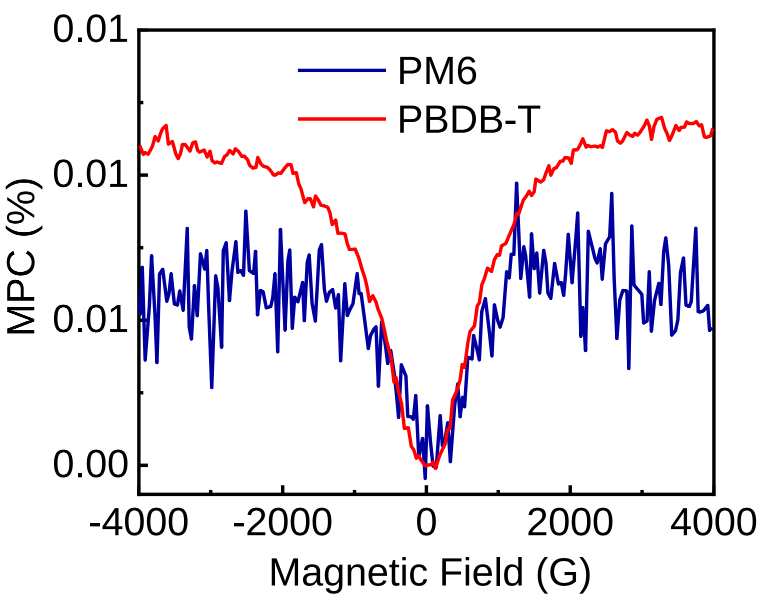

Figure S16. Magneto-photocurrent of PBDB-T and PM6 neat films at room temperature, the device structure is ITO/ZnO/prinstine film/MoO3/Ag..

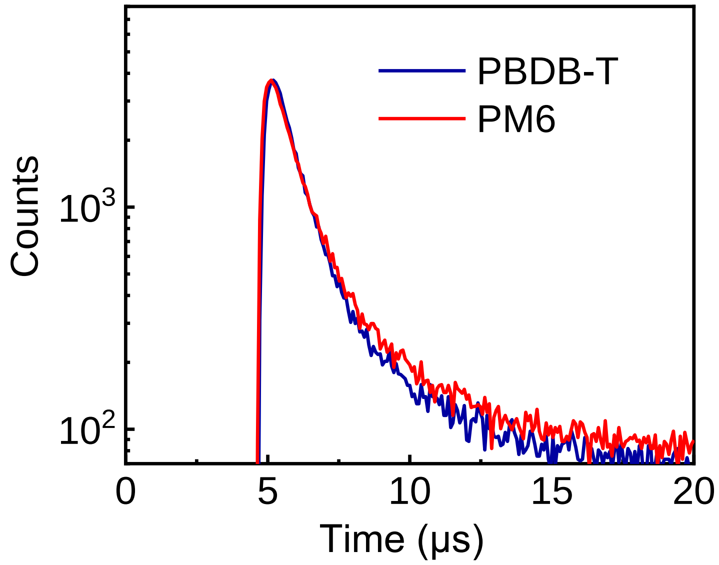

Figure S17. TPL Decay traces of PBDB-T and PM6 in 2-methylfuran solution at 77 K. The lifetime of PBDB-T and PM6 are evaluated to be 931 ns and 994 ns respectively.

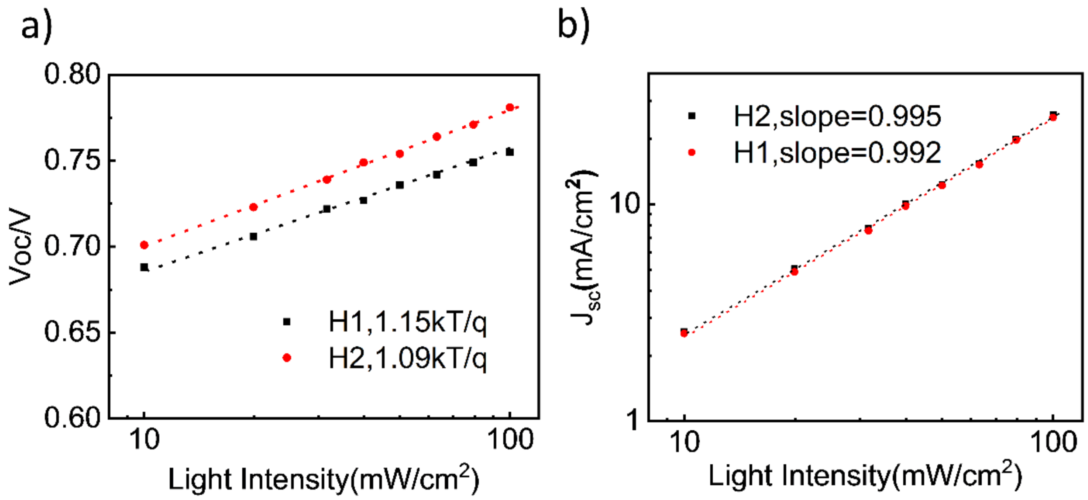

Figure S18. a) V_{OC} and b) J_{SC} dependence on the light intensity.

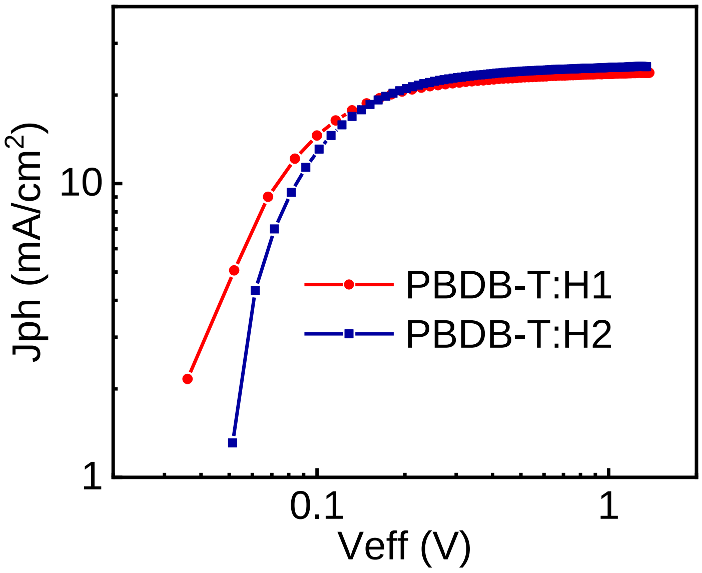

Figure S19. J_{ph}-V_{eff} plots for PBDB-T:H1 and PBDB-T:H2 devices.

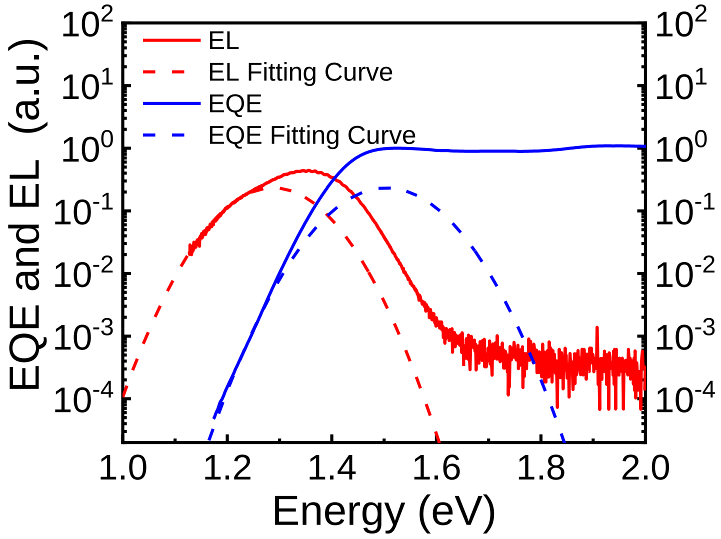

Figure S20. EL and EQE spectra of PBDB-T:H2 based devices.

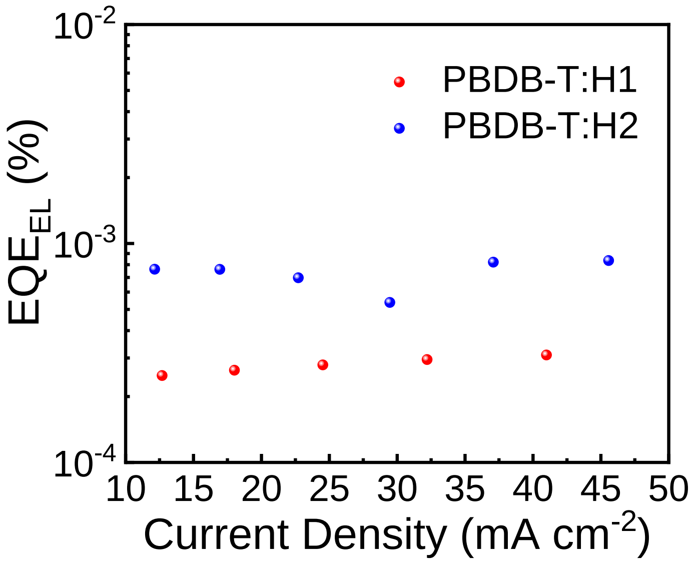

Figure S21. EL external quantum efficiencies at different injected current densities of the solar cells.

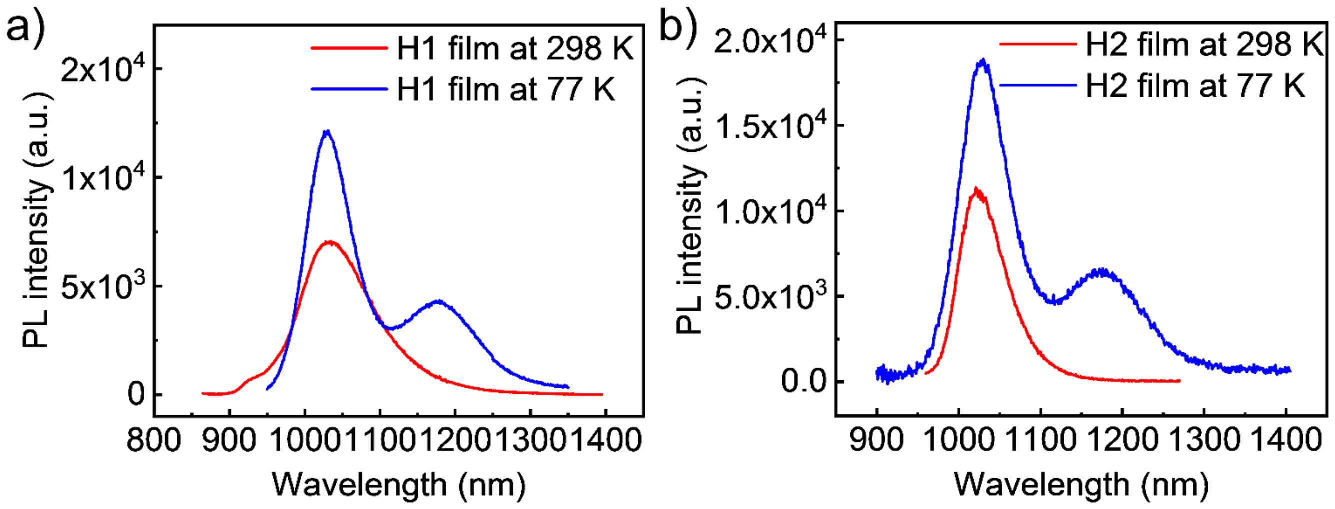

Figure S22. PL spectra of a) H1 and b) H2 film at 298 K and 77 K.

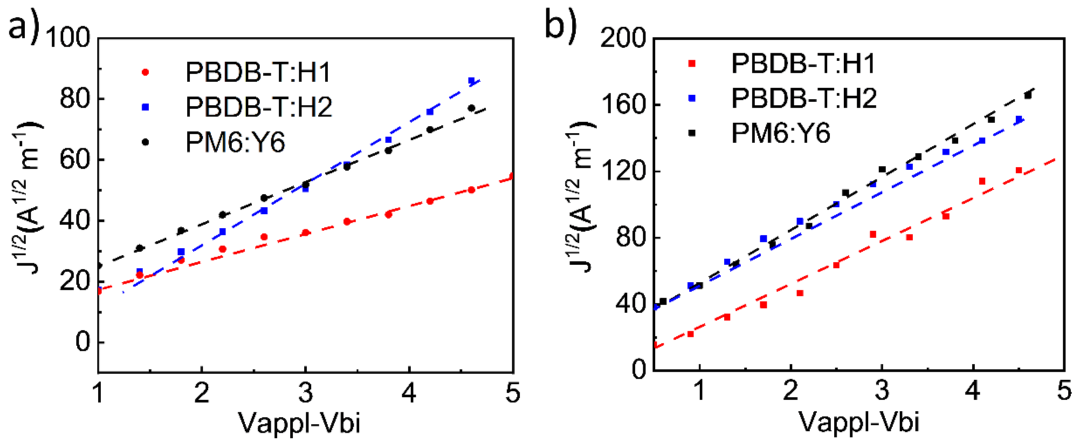

Figure S23. $J^{1/2}$-V plots for a) hole-only and b) electron-only devices for PBDB-T:H1, PBDB-T:H2 and PM6:Y6 devices.

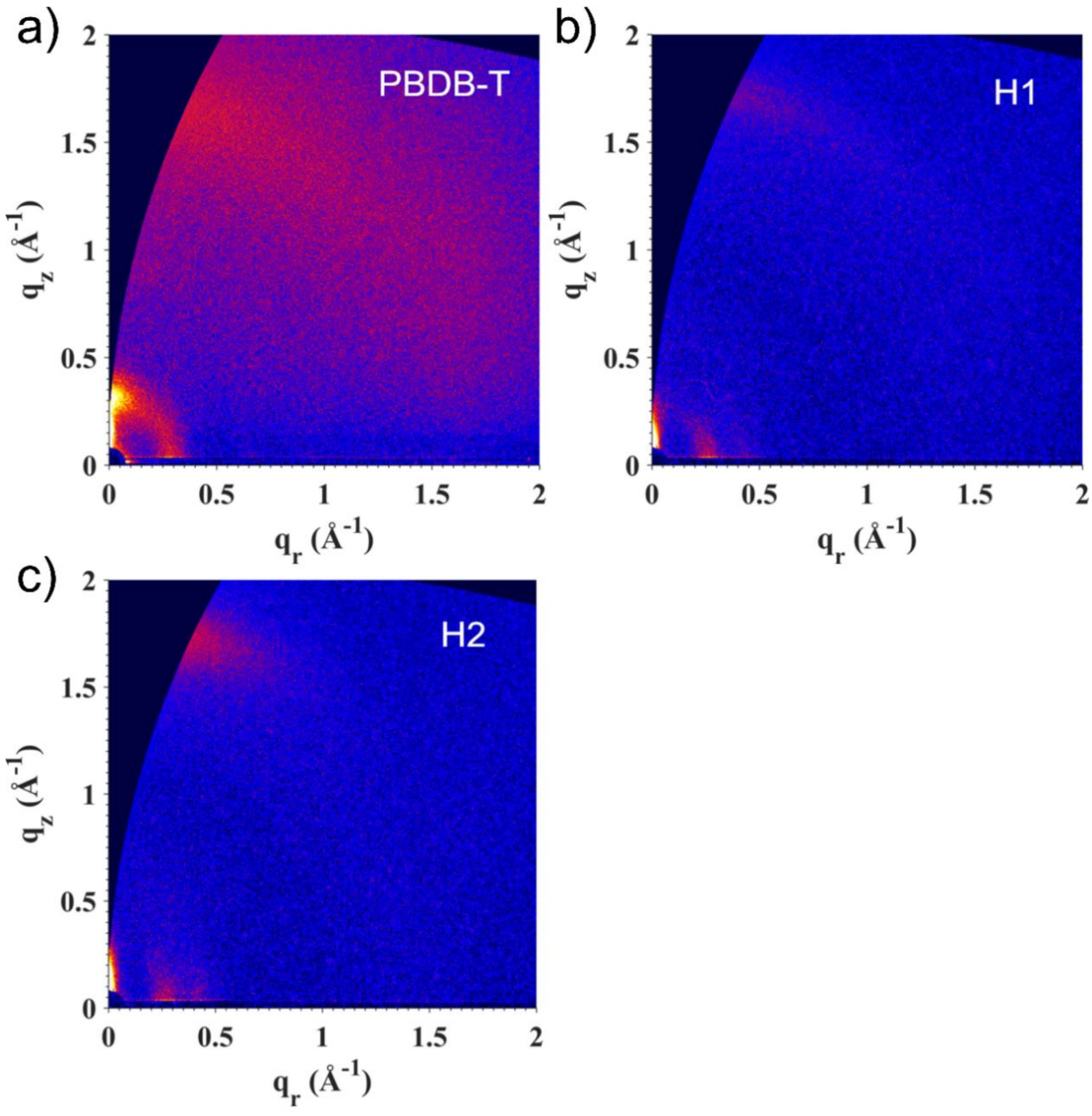

Figure S24. 2D GIWAXS patterns of a) PBDB-T, b) H1 and c) H2 pristine films.

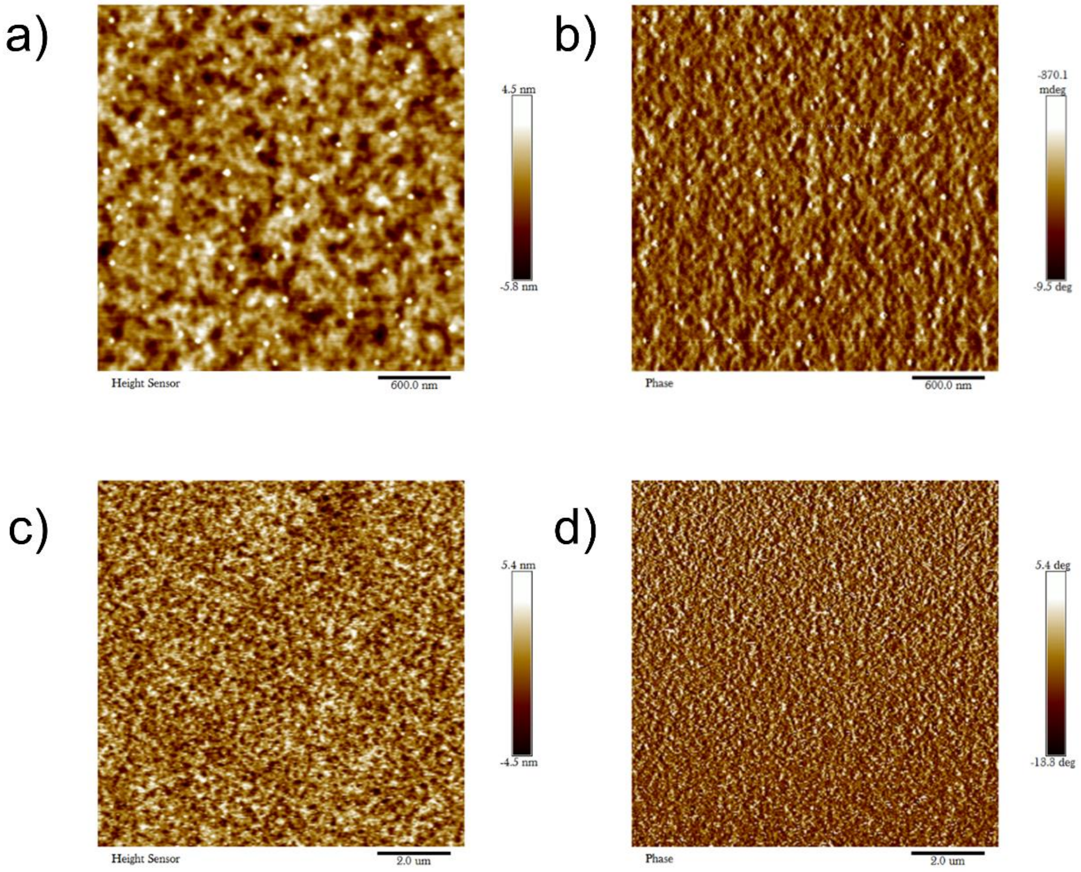

Figure S25. AFM height image of a) H1, c) H2 and phase image of b) H1, d) H2.

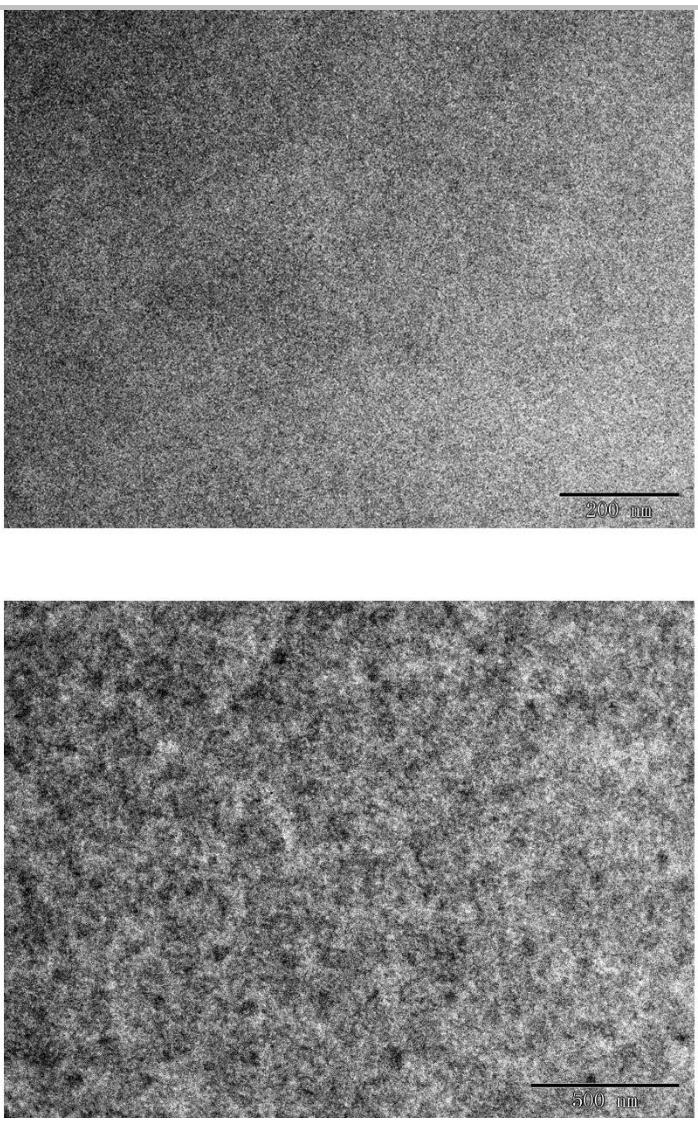

Figure S26. TEM images of a) H1 and b) H2.

Table S1. Excited states energy levels of all three isomers of H1 (naming H1-1, H1-2 and H1-3) calculated by Gaussian16 program at B3LYP/6-31G(d) level.

Excited States	H1-1 (eV)	H1-2 (eV)	H1-3 (eV)
S_1	1.7433	1.7608	1.7275
S_2	2.0852	2.1143	2.0555
S_3	2.3696	2.3627	2.3893
S_4	2.4278	2.4299	2.4138
S_5	2.4611	2.4486	2.4674
T_1	1.2462	1.2622	1.2324
T_2	1.5080	1.5320	1.4827
T_3	2.0180	2.0268	2.0065
T_4	2.0680	2.0818	2.0508
T_5	2.1172	2.1122	2.1286

Table S2. Excited states energy levels of all three isomers of H2 (naming H2-1, H2-2 and H2-3) calculated by Gaussian16 program at B3LYP/6-31G(d) level.

Excited States	H2-1 (eV)	H2-2 (eV)	H2-3 (eV)

S_1	1.7296	1.7346	1.7246
S_2	2.0667	2.0753	2.0580
S_3	2.3384	2.3357	2.3424
S_4	2.4077	2.4110	2.4029
S_5	2.4274	2.4238	2.4306
T_1	1.2375	1.2445	1.2310
T_2	1.4969	1.5078	1.4859
T_3	1.9894	1.9919	1.9867
T_4	2.0476	2.0501	2.0433
T_5	2.0918	2.0898	2.0958

Table S3. Excited states energy levels of Y6 calculated by Gaussian16 program at B3LYP/6-31G(d) level.

Excited States	Y6 (eV)
S_1	1.7355
S_2	2.0675
S_3	2.3394
S_4	2.4119
S_5	2.4287
T_1	1.2431
T_2	1.5040
T_3	1.9950
T_4	2.0513

Table S4. Spin orbit coupling constants of all three isomers of H1 calculated by Gaussian16 program at B3LYP/6-31G(d) level.[1]

Spin orbit coupling constant	H1-1 (cm-1)	H1-2 (cm-1)	H1-3 (cm-1)
$\langle S_1 \vert HSO \vert T_1 \rangle$	0.02103	0.02199	0.01907
$\langle S_1 \vert HSO \vert T_2 \rangle$	0.09101	0.09495	0.08697
$\langle S_1 \vert HSO \vert T_3 \rangle$	0.01771	0.01753	0.01362
$\langle S_1 \vert HSO \vert T_4 \rangle$	0.10119	0.11513	0.09970
$\langle S_1 \vert HSO \vert T_5 \rangle$	0.21664	0.13495	0.38825
$\langle S_2 \vert HSO \vert T_1 \rangle$	0.23905	0.24599	0.23248
$\langle S_2 \vert HSO \vert T_2 \rangle$	0.03196	0.03191	0.03050
$\langle S_2 \vert HSO \vert T_3 \rangle$	0.09591	0.09168	0.09842
$\langle S_2 \vert HSO \vert T_4 \rangle$	0.00621	0.00413	0.00335

Table S5. Spin orbit coupling constants of all three isomers of H2 calculated by Gaussian16 program at B3LYP/6-31G(d) level

Spin orbit coupling constant	H2-1 (cm-1)	H2-2 (cm-1)	H2-3 (cm-1)
$\langle S_1 \vert HSO \vert T_1 \rangle$	0.02086	0.02120	0.01888
$\langle S_1 \vert HSO \vert T_2 \rangle$	0.08950	0.09163	0.08822
$\langle S_1 \vert HSO \vert T_3 \rangle$	0.01315	0.00986	0.01278
$\langle S_1 \vert HSO \vert T_4 \rangle$	0.13017	0.14296	0.11848
$\langle S_1 \vert HSO \vert T_5 \rangle$	0.17551	0.13419	0.22309
$\langle S_2 \vert HSO \vert T_1 \rangle$	0.24719	0.25545	0.23906
$\langle S_2 \vert HSO \vert T_2 \rangle$	0.03239	0.03002	0.03439
$\langle S_2 \vert HSO \vert T_3 \rangle$	0.09382	0.09281	0.09494
$\langle S_2 \vert HSO \vert T_4 \rangle$	0.00461	0.00164	0.00504
$\langle S_2 \vert HSO \vert T_5 \rangle$	0.02064	0.02011	0.02149

Table S6. Spin orbit coupling constants of Y6 calculated by Gaussian16 program at B3LYP/6-31G(d) level

Spin orbit coupling constant	Y6
$\langle S_1 \vert HSO \vert T_1 \rangle$	0.01995

$<S_1\|HSO\|T_2>$	0.09034
$<S_1\|HSO\|T_3>$	0.01375
$<S_1\|HSO\|T_4>$	0.11913
$<S_1\|HSO\|T_5>$	0.18213
$<S_2\|HSO\|T_1>$	0.23819
$<S_2\|HSO\|T_2>$	0.03047
$<S_2\|HSO\|T_3>$	0.09089
$<S_2\|HSO\|T_4>$	0.00310
$<S_2\|HSO\|T_5>$	0.02458

Table S7. Detailed photovoltaic parameters of other OPV cells

Devices	V_{OC} (V)	J_{SC} (mA cm-2)	FF	PCE (%)
PM6:H1	0.85	19.97	0.54	9.17
PM6:H2	0.87	19.12	0.62	10.31
PBDB-T:Y6	0.73	24.59	0.56	9.88
PBDB-T:Y6 [a]	0.72	25.0	0.62	10.8

[a] The OSCs performance data was obtained from Ref. [2].

References

[1] X. Gao, S. Bai, D. Fazzi, T. Niehaus, M. Barbatti, W. Thiel, *J. Chem. Theory Comput.* 2017, *13*, 515-524.

[2] R. Wang, J. Yuan, R. Wang, G. Han, T. Huang, W. Huang, J. Xue, H. C. Wang, C. Zhang, C. Zhu, P. Cheng, D. Meng, Y. Yi, K. H. Wei, Y. Zou, Y. Yang, *Adv. Mater.* **2019**, *31*, 1904215.

[3] Frisch, M. J.; Trucks, G. W.; Schlegel, H. B.; Scuseria, G. E.; Robb, M. A.; Cheeseman, J. R.; Scalmani, G.; Barone, V.; Petersson, G. A.; Nakatsuji, H.; Li, X.; Caricato, M.; Marenich, A. V.; Bloino, J.; Janesko, B. G.; Gomperts, R.; Mennucci, B.; Hratchian, H. P.; Ortiz, J. V.; Izmaylov, A. F.; Sonnenberg, J. L.; Williams; Ding, F.; Lipparini, F.; Egidi, F.; Goings, J.; Peng, B.; Petrone, A.; Henderson, T.; Ranasinghe, D.; Zakrzewski, V. G.; Gao, J.; Rega, N.; Zheng, G.; Liang, W.; Hada, M.; Ehara, M.; Toyota, K.; Fukuda, R.; Hasegawa, J.; Ishida, M.; Nakajima, T.; Honda, Y.; Kitao, O.; Nakai, H.; Vreven, T.; Throssell, K.; Montgomery, Jr., J. A.; Peralta, J. E.; Ogliaro, F.; Bearpark, M. J.; Heyd, J. J.; Brothers, E. N.; Kudin, K. N.; Staroverov, V. N.; Keith, T. A.; Kobayashi, R.; Normand, J.; Raghavachari, K.; Rendell, A. P.; Burant, J. C.; Iyengar, S. S.; Tomasi, J.; Cossi, M.; Millam, J. M.; Klene, M.; Adamo, C.; Cammi, R.; Ochterski, J. W.; Martin, R. L.; Morokuma, K.; Farkas, O.; Foresman, J. B.; Fox, D. J. Gaussian 16; Gaussian, Inc.: Wallingford, CT, 2016.
